# Supplementary material for: Global terrestrial invasions: Where naturalised birds, mammals, and plants might spread next and what affects this process
Source: PLoS Biol. 2023 Nov 14;21(11):e3002361. doi: 10.1371/journal.pbio.3002361 (PMC10645288; doi:10.1371/journal.pbio.3002361)
Supplement: S1 Text — (DOCX) [file pbio.3002361.s001.docx]

**Supplementary methods**

We compared our datasets to GLONAF (plants), DAMA (mammals), and GAVIA (birds), which are the most comprehensive current assessments of naturalisations. Here we compare those datasets to the number of species in our dataset, including both the **initial** lists and the lists **after** filtering out naturalisations we could not confirm, naturalisations within the same biogeographic realm as the native range or on islands, casual aliens, taxonomically confusing species, subspecies, species with unconfirmed native ranges and migrants (most of which would be included in GLONAF, DAMA, and GAVIA). The published datasets do not provide coordinates of species locations, so we cannot know exactly how many species might be recorded in >5 10 arc-minute grid-cells (one of our thresholds for inclusion). Therefore, we calculated how many species are found in more than two geographic regions.

GLONAF [1] contains 11987 non-hybrid, naturalised taxa recorded to species level, with 6711 that are found in two or more TDWG4 regions (some of these may only be naturalised on islands). In comparison, the initial plant species list we compiled contained 2253 species, and after filtering we obtained 616 species.

DAMA [2] contains 230 species of mammals that have naturalised, with 139 found in more than two GADM level 1 administrative areas. 81% of the range polygons drawn with the data were on islands. In comparison, the initial mammal species we compiled (drawing on the same source used for the majority of DAMA species) contained 232 species, and after filtering we obtained 65 species. Given that so many DAMA locations are on islands, our mammal dataset would likely be very similar if we had initially used DAMA.

GAVIA [3] contains 971 species, with 284 of having established/breeding populations on a mainland, and 177 species being found in more than two localities. In comparison, the initial bird species list we compiled (from a prototype of GAVIA) contained 617 species, and after filtering we obtained 114 species. Therefore, our analysis is unlikely to have been changed substantially by using the more up to date GAVIA list.

1. van Kleunen M, Pyšek P, Dawson W, Essl F, Kreft H, Pergl J, et al. The Global Naturalized Alien Flora (GloNAF) database. Ecology. 2019;100(1):e02542. doi: <https://doi.org/10.1002/ecy.2542>.

2. Biancolini D, Vascellari V, Melone B, Blackburn TM, Cassey P, Scrivens SL, et al. DAMA: the global Distribution of Alien Mammals database. Ecology. 2021;102(11):e03474. doi: <https://doi.org/10.1002/ecy.3474>.

3. Dyer EE, Redding DW, Blackburn TM. The global avian invasions atlas, a database of alien bird distributions worldwide. Scientific Data. 2017;4(1):170041. doi: 10.1038/sdata.2017.41.
