# Supplementary material for: Global terrestrial invasions: Where naturalised birds, mammals, and plants might spread next and what affects this process
Source: PLoS Biol. 2023 Nov 14;21(11):e3002361. doi: 10.1371/journal.pbio.3002361 (PMC10645288; doi:10.1371/journal.pbio.3002361)
Supplement: S1 Table — “Median range size” is the median range size (calculated with minimum convex hull polygon) of all established naturalised species (measured in 1,000 km2). “Median potential range size” is the median area each species has available but has not colonised in each realm (measured in 1,000 km2). For each species, the total occupied area is divided by the total available area to return a range filling proportion. “Median proportion of filling” is the median range filling across all species in the given realm. (DOCX) [file pbio.3002361.s002.docx]

**Table S1:** Summary statistics of introduced species and range filling for all taxonomic groups across all biogeographic realms. “Median range size” is the median range size (calculated with minimum convex hull polygon) of all established naturalised species (measured in 1000 km^2^). “Median potential range size” is the median area each species has available but has not colonised in each realm (measured in 1000 km^2^). For each species the total occupied area is divided by the total available area to return a range filling proportion. “Median proportion of filling” is the median range filling across all species in the given realm.

|  | **Region** | **Number of introductions** | **Median Range Size (1000 km^2^)** | **Median Potential Range Size (1000 km^2^)** | **Median Range Filling Proportion** |
| --- | --- | --- | --- | --- | --- |
| **Plants** | GLOBAL | 484 | 441.45 | 4487.42 | 0.10 |
|  | Afrotropical | 19 | 145.75 | 6105.19 | 0.02 |
|  | Australian | 171 | 476.99 | 2739.75 | 0.15 |
|  | Nearctic | 174 | 887.27 | 7789.48 | 0.11 |
|  | Neotropical | 89 | 131.06 | 3625.80 | 0.34 |
|  | Oriental | 1 | 322.71 | 6260.12 | 0.05 |
|  | Palearctic (East) | 3 | 15.76 | 8579.61 | 0.002 |
|  | Palearctic (West) | 13 | 511.22 | 8824.98 | 0.07 |
|  | Saharo-Arabian | 4 | 71.80 | 9362.50 | 0.01 |
|  | Sino-Japanese | 10 | 145.48 | 2149.68 | 0.07 |
|  |  |  |  |  |  |
| **Birds** | GLOBAL | 50 | 112.12 | 5538.15 | 0.02 |
|  | Afrotropical | 2 | 989.46 | 21991.87 | 0.04 |
|  | Australian | 5 | 744.40 | 5879.41 | 0.10 |
|  | Nearctic | 23 | 87.26 | 5276.60 | 0.01 |
|  | Neotropical | 4 | 156.32 | 12814.93 | 0.01 |
|  | Palearctic (West) | 11 | 320.49 | 3780.78 | 0.10 |
|  | Panamanian | 3 | 103.24 | 916.98 | 0.10 |
|  | Saharo-Arabian | 2 | 44.79 | 6402.21 | 0.01 |
|  |  |  |  |  |  |
| **Mammals** | GLOBAL | 46 | 283.84 | 3102.15 | 0.11 |
|  | Afrotropical | 2 | 2967.62 | 14815.67 | 0.14 |
|  | Australian | 7 | 471.68 | 2529.87 | 0.59 |
|  | Nearctic | 12 | 258.85 | 4988.17 | 0.10 |
|  | Neotropical | 7 | 247.85 | 10819.92 | 0.02 |
|  | Palearctic (West) | 12 | 571.31 | 5933.23 | 0.10 |
|  | Panamanian | 4 | 191.94 | 642.81 | 0.21 |
|  | Saharo-Arabian | 2 | 76.96 | 2100.02 | 0.06 |
