# Supplementary material for: Global terrestrial invasions: Where naturalised birds, mammals, and plants might spread next and what affects this process
Source: PLoS Biol. 2023 Nov 14;21(11):e3002361. doi: 10.1371/journal.pbio.3002361 (PMC10645288; doi:10.1371/journal.pbio.3002361)
Supplement: S2 Table — This table presents results for the same model as in Table 1, but when only plants that have naturalised in >20 grid-cells were included. Estimates for parameters retained in the final model are given as the mean estimate of all posterior draws, with the 5% and 95% estimates as confidence intervals in parentheses. Parameter estimates are given as the linear slope of the logit link equation. When parameter estimates vary across realms, this is indicated by providing the names of the realms in which it varies (Aus = Australian, Nea = Nearctic, Neo = Neotropical). Model verification data are given for the final models, including sample size, DIC of the model, the effective number of parameters (pD), and correlation of the linear predictor against the link transformed response given as a pseudo R-squared. (DOCX) [file pbio.3002361.s003.docx]

**Table S2:** Sensitivity analysis of correlates of range filling for plants. This table presents results for the same model as in Table 1, but when only plants that have naturalised in > 20 grid-cells were included. Estimates for parameters retained in the final model are given as the mean estimate of all posterior draws, with the 5% and 95% estimates as confidence intervals in parentheses. Parameter estimates are given as the linear slope of the logit link equation. When parameter estimates vary across realms, this is indicated by providing the names of the realms in which it varies (Aus = Australian, Nea = Nearctic, Neo=Neotropical). Model verification data are given for the final models, including sample size, Deviance Information Criterion (DIC) of the model, the effective number of parameters (pD), and correlation of the linear predictor against the link transformed response given as a pseudo R-squared.

|  | **Model Parameter** | **Estimate** | **95% CI** | **Differences between realms?** | **Model Verification** | **Estimate** |
| --- | --- | --- | --- | --- | --- | --- |
| **Plants** | Intercept | -1.98 | (-1.25, -2.71) | Aus | Sample Size | 411 |
|  | Years since Introduction | 0.27 | (0.45, 0.11) |  | DIC | -738.13 |
|  | Days till Flowering (logged) | -0.16 | (0.03, -0.31) | Aus, Nea | pD | 16.13 |
|  | Local sampling effort | -0.21 | (0.53, -0.74) | Aus | Pseudo R-Squared | 0.31 |
