# Supplementary material for: Global terrestrial invasions: Where naturalised birds, mammals, and plants might spread next and what affects this process
Source: PLoS Biol. 2023 Nov 14;21(11):e3002361. doi: 10.1371/journal.pbio.3002361 (PMC10645288; doi:10.1371/journal.pbio.3002361)
Supplement: S3 Table — (DOCX) [file pbio.3002361.s004.docx]

**Table S3:** List of all plant species used in this study.

| *Abies concolor* | *Epilobium hirsutum* | *Pastinaca sativa* |
| --- | --- | --- |
| *Abies lasiocarpa* | *Epilobium parviflorum* | *Paulownia tomentosa* |
| *Abutilon theophrasti* | *Epiphyllum phyllanthus* | *Peganum harmala* |
| *Acacia dealbata* | *Equisetum arvense* | *Pennisetum ciliare* |
| *Acacia mearnsii* | *Eragrostis cilianensis* | *Pennisetum purpureum* |
| *Acacia melanoxylon* | *Eragrostis curvula* | *Pennisetum setaceum* |
| *Acer ginnala* | *Eragrostis echinochloidea* | *Phalaris arundinacea* |
| *Acer negundo* | *Eremopyrum triticeum* | *Phalaris minor* |
| *Acer platanoides* | *Erigeron speciosus* | *Phleum pratense* |
| *Acer pseudoplatanus* | *Erodium cicutarium* | *Phlox paniculata* |
| *Acer saccharinum* | *Eruca vesicaria* | *Phoenix canariensis* |
| *Achillea millefolium* | *Erysimum repandum* | *Phoenix dactylifera* |
| *Acroptilon repens* | *Eucalyptus camaldulensis* | *Physalis virginiana* |
| *Adenanthera pavonina* | *Eugenia uniflora* | *Picea abies* |
| *Aegilops cylindrica* | *Euonymus fortunei* | *Picea engelmannii* |
| *Aegopodium podagraria* | *Euphorbia cyparissias* | *Picea pungens* |
| *Agropyron cristatum* | *Euphorbia dendroides* | *Pinus contorta* |
| *Agrostis capillaris* | *Euphorbia esula* | *Pinus ponderosa* |
| *Agrostis gigantea* | *Euphorbia mellifera* | *Pinus sylvestris* |
| *Agrostis scabra* | *Euphorbia myrsinites* | *Pistacia vera* |
| *Ailanthus altissima* | *Euphorbia tirucalli* | *Pistia stratiotes* |
| *Akebia quinata* | *Euryops multifidus* | *Plantago lanceolata* |
| *Albizia julibrissin* | *Festuca arundinacea* | *Plantago major* |
| *Albizia lebbeck* | *Festuca ovina* | *Poa annua* |
| *Alhagi maurorum* | *Festuca trachyphylla* | *Poa bulbosa* |
| *Alliaria petiolata* | *Flacourtia indica* | *Poa compressa* |
| *Allium vineale* | *Foeniculum vulgare* | *Poa nemoralis* |
| *Alnus glutinosa* | *Fragaria vesca* | *Poa palustris* |
| *Aloe arborescens* | *Fragaria virginiana* | *Poa pratensis* |
| *Aloe maculata* | *Frangula alnus* | *Poa trivialis* |
| *Alopecurus geniculatus* | *Fraxinus americana* | *Podalyria sericea* |
| *Alopecurus myosuroides* | *Fraxinus pennsylvanica* | *Polygonum arenastrum* |
| *Alopecurus pratensis* | *Gaillardia aristata* | *Polygonum caespitosum* |
| *Alternanthera philoxeroides* | *Galanthus elwesii* | *Polygonum perfoliatum* |
| *Alyssum alyssoides* | *Galanthus nivalis* | *Polygonum persicaria* |
| *Alyssum desertorum* | *Galeopsis tetrahit* | *Polypogon monspeliensis* |
| *Amaranthus albus* | *Geranium molle* | *Polypogon viridis* |
| *Amaranthus blitoides* | *Geranium pusillum* | *Poncirus trifoliata* |
| *Ambrosia artemisiifolia* | *Geum macrophyllum* | *Pontederia cordata* |
| *Ambrosia trifida* | *Glaucium flavum* | *Populus alba* |
| *Amelanchier alnifolia* | *Glechoma hederacea* | *Populus deltoides* |
| *Amorpha fruticosa* | *Gleditsia triacanthos* | *Populus nigra* |
| *Ampelopsis brevipedunculata* | *Glossostigma cleistanthum* | *Portulacaria afra* |
| *Amsinckia menziesii* | *Glyceria maxima* | *Potamogeton crispus* |
| *Anthemis arvensis* | *Glyceria striata* | *Potentilla argentea* |
| *Anthemis cotula* | *Grevillea robusta* | *Potentilla pensylvanica* |
| *Anthoxanthum odoratum* | *Grindelia squarrosa* | *Potentilla recta* |
| *Anthriscus caucalis* | *Gypsophila paniculata* | *Potentilla rivalis* |
| *Anthriscus sylvestris* | *Harrisia martinii* | *Prunus lusitanica* |
| *Antigonon leptopus* | *Harrisia tortuosa* | *Prunus serotina* |
| *Apium graveolens* | *Hedera helix* | *Prunus virginiana* |
| *Aquilegia vulgaris* | *Hemarthria altissima* | *Pseudotsuga menziesii* |
| *Aralia chinensis* | *Hemerocallis fulva* | *Psidium cattleianum* |
| *Araucaria araucana* | *Hesperis matronalis* | *Psidium guajava* |
| *Arctium minus* | *Hibiscus syriacus* | *Pteris vittata* |
| *Ardisia crenata* | *Hiptage benghalensis* | *Pterocarpus indicus* |
| *Ardisia elliptica* | *Holcus lanatus* | *Puccinellia distans* |
| *Armeniaca vulgaris* | *Hordeum jubatum* | *Pyracantha koidzumii* |
| *Arnica chamissonis* | *Hordeum marinum* | *Pyrostegia venusta* |
| *Arrhenatherum elatius* | *Hordeum murinum* | *Pyrus calleryana* |
| *Artemisia absinthium* | *Hydrilla verticillata* | *Ranunculus cymbalaria* |
| *Artemisia campestris* | *Hydrocharis morsus-ranae* | *Ranunculus ficaria* |
| *Artemisia dracunculus* | *Hydrocleys nymphoides* | *Ranunculus repens* |
| *Artemisia vulgaris* | *Hylocereus undatus* | *Ranunculus sceleratus* |
| *Arundo donax* | *Hymenachne amplexicaulis* | *Rapistrum rugosum* |
| *Asparagus officinalis* | *Hyoscyamus niger* | *Rhamnus cathartica* |
| *Asphodelus fistulosus* | *Hypericum perforatum* | *Rhus glabra* |
| *Asystasia gangetica* | *Hypochaeris radicata* | *Ribes aureum* |
| *Avena barbata* | *Impatiens capensis* | *Robinia pseudoacacia* |
| *Avena fatua* | *Imperata cylindrica* | *Rorippa amphibia* |
| *Azolla caroliniana* | *Ipomoea aquatica* | *Rorippa nasturtium-aquaticum* |
| *Azolla filiculoides* | *Ipomoea purpurea* | *Rorippa palustris* |
| *Azolla pinnata* | *Iris pseudacorus* | *Rorippa sylvestris* |
| *Babiana angustifolia* | *Isatis tinctoria* | *Rosa multiflora* |
| *Babiana stricta* | *Jacaranda mimosifolia* | *Rosa rugosa* |
| *Barbarea vulgaris* | *Juglans nigra* | *Rotala rotundifolia* |
| *Bauhinia variegata* | *Juglans regia* | *Rubus allegheniensis* |
| *Begonia cucullata* | *Juncus bufonius* | *Rubus idaeus* |
| *Berberis thunbergii* | *Juncus compressus* | *Rubus parviflorus* |
| *Berberis vulgaris* | *Juncus ensifolius* | *Rubus phoenicolasius* |
| *Berteroa incana* | *Juncus gerardii* | *Rumex acetosa* |
| *Beta macrocarpa* | *Juncus inflexus* | *Rumex acetosella* |
| *Betula pendula* | *Juncus tenuis* | *Rumex crispus* |
| *Bidens pilosa* | *Juniperus virginiana* | *Rumex longifolius* |
| *Brassica nigra* | *Kalanchoe pinnata* | *Rumex obtusifolius* |
| *Brassica tournefortii* | *Khaya senegalensis* | *Saccharum ravennae* |
| *Briza maxima* | *Kochia scoparia* | *Sagina procumbens* |
| *Bromus arvensis* | *Koeleria macrantha* | *Sagittaria graminea* |
| *Bromus carinatus* | *Lactuca serriola* | *Sagittaria montevidensis* |
| *Bromus catharticus* | *Lamium amplexicaule* | *Sagittaria rigida* |
| *Bromus hordeaceus* | *Lampranthus glaucus* | *Salix alba* |
| *Bromus inermis* | *Lantana camara* | *Salix purpurea* |
| *Bromus japonicus* | *Lathyrus latifolius* | *Salsola kali* |
| *Bromus racemosus* | *Leonurus cardiaca* | *Salvia aethiopis* |
| *Bromus secalinus* | *Lepidium densiflorum* | *Salvinia minima* |
| *Bromus tectorum* | *Lepidium latifolium* | *Sambucus racemosa* |
| *Broussonetia papyrifera* | *Lepidium perfoliatum* | *Sanguisorba minor* |
| *Butomus umbellatus* | *Lepidium virginicum* | *Saponaria officinalis* |
| *Cabomba caroliniana* | *Lespedeza cuneata* | *Sarracenia purpurea* |
| *Caesalpinia gilliesii* | *Leucaena leucocephala* | *Schefflera actinophylla* |
| *Callitriche stagnalis* | *Leucanthemum vulgare* | *Schinus terebinthifolius* |
| *Camelina microcarpa* | *Leymus arenarius* | *Schismus barbatus* |
| *Campanula rapunculoides* | *Ligustrum japonicum* | *Sedum acre* |
| *Capsella bursa-pastoris* | *Ligustrum lucidum* | *Senecio jacobaea* |
| *Cardamine impatiens* | *Ligustrum ovalifolium* | *Senecio sylvaticus* |
| *Cardaria draba* | *Ligustrum sinense* | *Senecio vulgaris* |
| *Cardaria pubescens* | *Ligustrum vulgare* | *Senna pendula* |
| *Carduus acanthoides* | *Limnobium laevigatum* | *Sequoia sempervirens* |
| *Carduus crispus* | *Linaria vulgaris* | *Sequoiadendron giganteum* |
| *Carduus nutans* | *Linum perenne* | *Sesbania punicea* |
| *Carduus pycnocephalus* | *Lolium multiflorum* | *Setaria faberi* |
| *Carduus tenuiflorus* | *Lolium perenne* | *Setaria viridis* |
| *Carex disticha* | *Lonicera involucrata* | *Silene antirrhina* |
| *Carex flacca* | *Lonicera japonica* | *Silene noctiflora* |
| *Carum carvi* | *Lonicera maackii* | *Silene vulgaris* |
| *Casuarina cunninghamiana* | *Lonicera tatarica* | *Silphium perfoliatum* |
| *Casuarina equisetifolia* | *Lonicera xylosteum* | *Silybum marianum* |
| *Cedrela odorata* | *Lotus corniculatus* | *Sinapis arvensis* |
| *Celastrus orbiculatus* | *Ludwigia grandiflora* | *Sisymbrium altissimum* |
| *Cenchrus longispinus* | *Ludwigia peploides* | *Sisymbrium irio* |
| *Centaurea calcitrapa* | *Ludwigia peruviana* | *Sisymbrium loeselii* |
| *Centaurea cyanus* | *Lupinus polyphyllus* | *Sisymbrium officinale* |
| *Centaurea diffusa* | *Luzula luzuloides* | *Sisyrinchium angustifolium* |
| *Centaurea jacea* | *Lycopus europaeus* | *Sisyrinchium montanum* |
| *Centaurea melitensis* | *Lygodium japonicum* | *Solanum carolinense* |
| *Centaurea solstitialis* | *Lygodium microphyllum* | *Solanum dulcamara* |
| *Cerastium arvense* | *Lysimachia nummularia* | *Solanum nigrum* |
| *Cerastium fontanum* | *Lysimachia vulgaris* | *Solanum torvum* |
| *Ceratocephala testiculata* | *Lythrum salicaria* | *Solanum triflorum* |
| *Cereus hildmannianus* | *Macfadyena unguis-cati* | *Solanum viarum* |
| *Chamaecyparis lawsoniana* | *Maclura pomifera* | *Solidago canadensis* |
| *Chelidonium majus* | *Maianthemum stellatum* | *Solidago gigantea* |
| *Chenopodium album* | *Malcolmia africana* | *Solidago sempervirens* |
| *Chenopodium opulifolium* | *Malva neglecta* | *Sonchus arvensis* |
| *Chondrilla juncea* | *Malva parviflora* | *Sonchus asper* |
| *Chorispora tenella* | *Marrubium vulgare* | *Sonchus oleraceus* |
| *Cichorium intybus* | *Marsilea quadrifolia* | *Sorghum bicolor* |
| *Cinnamomum camphora* | *Medicago lupulina* | *Sorghum halepense* |
| *Cirsium arvense* | *Medicago minima* | *Sparaxis tricolor* |
| *Cirsium palustre* | *Medicago polymorpha* | *Spartina alterniflora* |
| *Cirsium vulgare* | *Medicago sativa* | *Spartina anglica* |
| *Clematis orientalis* | *Melaleuca quinquenervia* | *Spartina densiflora* |
| *Clematis terniflora* | *Melia azedarach* | *Spartina patens* |
| *Coleonema pulchrum* | *Melilotus indicus* | *Spathoglottis plicata* |
| *Collomia grandiflora* | *Melilotus officinalis* | *Sphagneticola trilobata* |
| *Collomia linearis* | *Melinis minutiflora* | *Spiraea japonica* |
| *Colocasia esculenta* | *Mentha aquatica* | *Spirodela punctata* |
| *Commelina diffusa* | *Mentha pulegium* | *Sporobolus cryptandrus* |
| *Conium maculatum* | *Mentha spicata* | *Sporobolus vaginiflorus* |
| *Conringia orientalis* | *Merremia tuberosa* | *Stellaria media* |
| *Convolvulus arvensis* | *Mesembryanthemum crystallinum* | *Stenocereus griseus* |
| *Conyza canadensis* | *Mesembryanthemum nodiflorum* | *Stuckenia vaginata* |
| *Cornus sericea* | *Microstegium vimineum* | *Swietenia macrophylla* |
| *Coronilla varia* | *Miscanthus sacchariflorus* | *Symphoricarpos albus* |
| *Cortaderia selloana* | *Miscanthus sinensis* | *Syringa vulgaris* |
| *Cotula coronopifolia* | *Monarda didyma* | *Syzygium cumini* |
| *Crepis capillaris* | *Monochoria vaginalis* | *Syzygium jambos* |
| *Cupaniopsis anacardioides* | *Morus alba* | *Taeniatherum caput-medusae* |
| *Cupressus macrocarpa* | *Murdannia keisak* | *Tamarix gallica* |
| *Cyathea cooperi* | *Myosotis arvensis* | *Tanacetum vulgare* |
| *Cycas revoluta* | *Myosotis scorpioides* | *Taxus cuspidata* |
| *Cyclamen coum* | *Myosotis sylvatica* | *Tecoma stans* |
| *Cyclamen hederifolium* | *Myosoton aquaticum* | *Thlaspi arvense* |
| *Cyclamen repandum* | *Myriophyllum aquaticum* | *Torilis japonica* |
| *Cylindropuntia imbricata* | *Myriophyllum heterophyllum* | *Tradescantia fluminensis* |
| *Cylindropuntia kleiniae* | *Myriophyllum spicatum* | *Tradescantia spathacea* |
| *Cylindropuntia spinosior* | *Najas graminea* | *Tragopogon porrifolius* |
| *Cylindropuntia tunicata* | *Najas minor* | *Tragopogon pratensis* |
| *Cynara cardunculus* | *Narcissus pseudonarcissus* | *Trapa natans* |
| *Cynodon dactylon* | *Nasturtium officinale* | *Triadica sebifera* |
| *Cynoglossum officinale* | *Nelumbo nucifera* | *Tribulus terrestris* |
| *Cyperus difformis* | *Nemesia strumosa* | *Trifolium arvense* |
| *Cyperus prolifer* | *Nepeta cataria* | *Trifolium campestre* |
| *Cyperus rotundus* | *Nerium oleander* | *Trifolium dubium* |
| *Cytisus scoparius* | *Nicotiana glauca* | *Trifolium hybridum* |
| *Dactylis glomerata* | *Nymphaea ampla* | *Trifolium pratense* |
| *Dalbergia sissoo* | *Nymphaea lotus* | *Trifolium repens* |
| *Datura stramonium* | *Nymphaea odorata* | *Tussilago farfara* |
| *Datura wrightii* | *Nymphoides peltata* | *Typha latifolia* |
| *Daucus carota* | *Oeceoclades maculata* | *Ulex europaeus* |
| *Delonix regia* | *Oenothera biennis* | *Ulmus pumila* |
| *Descurainia pinnata* | *Onopordum acanthium* | *Urtica dioica* |
| *Descurainia sophia* | *Opuntia aurantiaca* | *Utricularia gibba* |
| *Dianthus armeria* | *Opuntia elata* | *Valeriana officinalis* |
| *Dietes bicolor* | *Opuntia humifusa* | *Verbascum blattaria* |
| *Digitalis grandiflora* | *Opuntia leucotricha* | *Verbascum thapsus* |
| *Digitalis lutea* | *Opuntia microdasys* | *Verbascum virgatum* |
| *Digitalis purpurea* | *Opuntia monacantha* | *Verbena bonariensis* |
| *Digitaria sanguinalis* | *Opuntia phaeacantha* | *Veronica anagallis-aquatica* |
| *Dioscorea bulbifera* | *Opuntia polyacantha* | *Veronica arvensis* |
| *Dipsacus fullonum* | *Opuntia puberula* | *Veronica beccabunga* |
| *Dipsacus laciniatus* | *Opuntia robusta* | *Veronica peregrina* |
| *Dracocephalum parviflorum* | *Opuntia streptacantha* | *Vicia cracca* |
| *Duchesnea indica* | *Opuntia stricta* | *Vicia sativa* |
| *Echinochloa colona* | *Opuntia tomentosa* | *Vicia villosa* |
| *Echinochloa crus-galli* | *Ornithogalum umbellatum* | *Vinca major* |
| *Echium plantagineum* | *Ottelia alismoides* | *Vinca minor* |
| *Egeria densa* | *Oxalis stricta* | *Vincetoxicum nigrum* |
| *Eichhornia crassipes* | *Pachysandra terminalis* | *Vulpia bromoides* |
| *Elaeagnus angustifolia* | *Paederia foetida* | *Wisteria sinensis* |
| *Elaeagnus pungens* | *Panicum antidotale* | *Woodwardia radicans* |
| *Elaeagnus umbellata* | *Panicum repens* | *Xanthium strumarium* |
| *Elsholtzia ciliata* | *Parthenocissus quinquefolia* | *Zeuxine strateumatica* |
| *Elymus canadensis* | *Paspalum notatum* | *Zizania aquatica* |
| *Epilobium brachycarpum* | *Paspalum urvillei* |  |
| *Epilobium ciliatum* | *Passiflora foetida* |  |
