# Supplementary material for: Global terrestrial invasions: Where naturalised birds, mammals, and plants might spread next and what affects this process
Source: PLoS Biol. 2023 Nov 14;21(11):e3002361. doi: 10.1371/journal.pbio.3002361 (PMC10645288; doi:10.1371/journal.pbio.3002361)
Supplement: S4 Table — (DOCX) [file pbio.3002361.s005.docx]

**Table S4:** List of all bird species used in this study.

| *Acridotheres cristatellus* | *Chrysolophus amherstiae* | *Molothrus bonariensis* |
| --- | --- | --- |
| *Acridotheres fuscus* | *Chrysolophus pictus* | *Mycteria leucocephala* |
| *Acridotheres ginginianus* | *Colinus virginianus* | *Myiopsitta monachus* |
| *Acridotheres grandis* | *Copsychus malabaricus* | *Nandayus nenday* |
| *Acridotheres javanicus* | *Copsychus saularis* | *Neochmia temporalis* |
| *Acridotheres tristis* | *Corvus splendens* | *Nesoenas picturata* |
| *Agapornis fischeri* | *Coturnix ypsilophora* | *Numida meleagris* |
| *Agapornis personatus* | *Crotophaga ani* | *Nymphicus hollandicus* |
| *Agapornis roseicollis* | *Cygnus atratus* | *Padda oryzivora* |
| *Alectoris barbara* | *Dacelo novaeguineae* | *Paroaria capitata* |
| *Alectoris graeca* | *Estrilda astrild* | *Paroaria coronata* |
| *Amandava amandava* | *Estrilda caerulescens* | *Pavo cristatus* |
| *Amazona albifrons* | *Estrilda melpoda* | *Phasianus colchicus* |
| *Amazona amazonica* | *Estrilda troglodytes* | *Pica pica* |
| *Amazona autumnalis* | *Euplectes afer* | *Platycercus eximius* |
| *Amazona finschi* | *Euplectes franciscanus* | *Ploceus cucullatus* |
| *Amazona oratrix* | *Forpus passerinus* | *Ploceus manyar* |
| *Amazona viridigenalis* | *Foudia madagascariensis* | *Ploceus melanocephalus* |
| *Aplonis panayensis* | *Francolinus erckelii* | *Poicephalus senegalus* |
| *Ara ararauna* | *Francolinus francolinus* | *Porphyrio porphyrio* |
| *Ara severus* | *Francolinus pondicerianus* | *Psittacula cyanocephala* |
| *Aratinga erythrogenys* | *Garrulax canorus* | *Psittacula eupatria* |
| *Aratinga holochlora* | *Geopelia striata* | *Psittacula krameri* |
| *Aratinga mitrata* | *Gracula religiosa* | *Pterocles exustus* |
| *Aratinga nana* | *Gymnorhina tibicen* | *Pycnonotus aurigaster* |
| *Athene noctua* | *Leiothrix lutea* | *Pycnonotus cafer* |
| *Bambusicola thoracicus* | *Lonchura atricapilla* | *Pycnonotus jocosus* |
| *Bonasa umbellus* | *Lonchura cantans* | *Serinus canaria* |
| *Brotogeris chiriri* | *Lonchura castaneothorax* | *Serinus mozambicus* |
| *Brotogeris versicolurus* | *Lonchura malabarica* | *Sicalis flaveola* |
| *Cacatua galerita* | *Lonchura malacca* | *Streptopelia chinensis* |
| *Cacatua sanguinea* | *Lonchura punctulata* | *Streptopelia decaocto* |
| *Callipepla californica* | *Lophura ignita* | *Streptopelia senegalensis* |
| *Callipepla gambelii* | *Lophura leucomelanos* | *Streptopelia tranquebarica* |
| *Cardinalis cardinalis* | *Lophura nycthemera* | *Struthio camelus* |
| *Cereopsis novaehollandiae* | *Lyrurus tetrix* | *Syrmaticus reevesii* |
| *Chloebia gouldiae* | *Meleagris gallopavo* | *Taeniopygia guttata* |
| *Chloephaga picta* | *Menura novaehollandiae* | *Vidua macroura* |
