# Supplementary material for: Global terrestrial invasions: Where naturalised birds, mammals, and plants might spread next and what affects this process
Source: PLoS Biol. 2023 Nov 14;21(11):e3002361. doi: 10.1371/journal.pbio.3002361 (PMC10645288; doi:10.1371/journal.pbio.3002361)
Supplement: S5 Table — (DOCX) [file pbio.3002361.s006.docx]

**Table S5:** List of all mammal species used in this study.

| *Aepyceros melampus* | *Macropus rufogriseus* | *Pseudocheirus peregrinus* |
| --- | --- | --- |
| *Ammotragus lervia* | *Martes martes* | *Rattus argentiventer* |
| *Antilope cervicapra* | *Microtus arvalis* | *Rattus exulans* |
| *Atelerix algirus* | *Muntiacus reevesi* | *Rattus nitidus* |
| *Axis axis* | *Mus musculus* | *Rattus norvegicus* |
| *Bandicota indica* | *Mustela erminea* | *Rattus praetor* |
| *Boselaphus tragocamelus* | *Mustela nivalis* | *Rattus rattus* |
| *Cervus elaphus* | *Mustela putorius* | *Rattus tanezumi* |
| *Cervus nippon* | *Mustela sibirica* | *Rupicapra rupicapra* |
| *Crocidura russula* | *Myocastor coypus* | *Rusa unicolor* |
| *Crocidura suaveolens* | *Myodes glareolus* | *Sciurus carolinensis* |
| *Dama dama* | *Neovison vison* | *Sciurus niger* |
| *Didelphis marsupialis* | *Nyctereutes procyonoides* | *Sciurus vulgaris* |
| *Erinaceus europaeus* | *Odocoileus virginianus* | *Spilocuscus maculatus* |
| *Genetta genetta* | *Ondatra zibethicus* | *Suncus murinus* |
| *Glis glis* | *Oreamnos americanus* | *Sus scrofa* |
| *Herpestes ichneumon* | *Oryctolagus cuniculus* | *Sylvilagus floridanus* |
| *Herpestes javanicus* | *Ovibos moschatus* | *Tamias sibiricus* |
| *Hydropotes inermis* | *Paguma larvata* | *Tamiasciurus hudsonicus* |
| *Hystrix cristata* | *Petaurus breviceps* | *Vulpes lagopus* |
| *Lepus europaeus* | *Phascolarctos cinereus* | *Vulpes vulpes* |
| *Lepus timidus* | *Procyon lotor* |  |
