# Supplementary material for: Global terrestrial invasions: Where naturalised birds, mammals, and plants might spread next and what affects this process
Source: PLoS Biol. 2023 Nov 14;21(11):e3002361. doi: 10.1371/journal.pbio.3002361 (PMC10645288; doi:10.1371/journal.pbio.3002361)
Supplement: S6 Table — Unless otherwise stated, plant sources were accessed June 2017, bird and mammal sources were accessed September 2017. (DOCX) [file pbio.3002361.s007.docx]

**Table S6:** List of sources used to classify populations as native or naturalised. Unless otherwise stated plant sources were accessed June 2017, bird and mammal sources were accessed September 2017.

| Taxon | Citation |
| --- | --- |
| Plants | Administración de Parques Nacionales, Argentina (2019) Sistema de Información de Biodiversidad https://sib.gob.ar/#!/ |
|  | African Plant Database (version 3.4.0). Conservatoire et Jardin botaniques de la Ville de Genève and South African National Biodiversity Institute, Pretoria. http://www.ville-ge.ch/musinfo/bd/cjb/africa/. |
|  | Anton, A.M. & Zuloaga, F.O. (eds.) (2012). Brassicaceae. Flora Argentina. Estudio Sigma, Buenos Aires. Vol. 8, pp. 1-273. http://buscador.floraargentina.edu.ar/ |
|  | Atlas of Living Australia (2019) website at http://www.ala.org.au. |
|  | Bernal, R., S.R. Gradstein & M. Celis (eds.). 2019. Catálogo de plantas y líquenes de Colombia. Instituto de Ciencias Naturales, Universidad Nacional de Colombia, Bogotá. http://catalogoplantasdecolombia.unal.edu.co |
|  | Botanical Society of Britain and Ireland (2019). Flora of Northern Island. http://www.habitas.org.uk/flora/index.html |
|  | Breitwieser I., Brownsey P.J.; Heenan P.B., Nelson W.A., Wilton A.D. eds. (2010) Flora of New Zealand Online. Accessed at www.nzflora.info, |
|  | Brouillet, L., F. Coursol, S.J. Meades, M. Favreau, M. Anions, P. Bélisle & P. Desmet (2010-). VASCAN, the Database of Vascular Plants of Canada. http://data.canadensys.net/vascan/ |
|  | Burnham, R.J. (2008-2014). "CLIMBERS: Censusing Lianas in Mesic Biomes of Eastern Regiond." http://climbers.lsa.umich.edu |
|  | CABI (2019). Invasive Species Compendium. Wallingford, UK: CAB International. www.cabi.org/isc. |
|  | Center for Invasive Species and Ecosystem Health (2019). Invasive.org database. https://www.invasive.org/ |
|  | Chacón E. (2019) Flora de Costa Rica http://floracostaricensis.myspecies.info/ |
|  | China Checklist (2019). Checklist of the Vascular Plants of China. Missouri Botanical Garden, St. Louis, U.S.A. http://www.tropicos.org/NameSearch.aspx?projectid=8 |
|  | DAISIE (Delivering Alien Invasive Species Inventories for Europe) (2009). Handbook of alien species in Europe. Invading Nature - Springer Series in Invasion Ecology, 3. Springer: Dordrecht . ISBN 978-1-4020-8279-5. xxviii, 399 pp. http://www.europe-aliens.org/default.do |
|  | Danin, A. & O. Fragman- Sapir. (2016-). Flora of Israel Online. http://flora.org.il/en/plants/ |
|  | De Egea, J., Mereles, F., del Carmen Pena-Chocarro, M., & Céspedes, G. (2016). Checklist for the crop weeds of Paraguay. PhytoKeys, (73), 13. |
|  | Department of Parks and Wildlife (DPaW), Western Australian Government (2008): FloraBase - WA flora database. https://florabase.dpaw.wa.gov.au/ |
|  | Driver, M., Raimondo, D., Maze, K., Pfab, M.F. and Helme, N.A. 2009. Applications of the Red List for conservation practitioners. In: D. Raimondo, L. Von Staden, W. Foden, J.E. Victor, N.A. Helme, R.C. Turner, D.A. Kamundi and P.A. Manyama (eds). Red List of South African Plants. Strelitzia 25:41-52. South African National Biodiversity Institute, Pretoria. http://redlist.sanbi.org/index.php |
|  | Ecuador Catalogue (2019). Catalogue of the Vascular Plants of Ecuador. Missouri Botanical Garden, St. Louis, U.S.A. & Antananarivo, Madagascar http://www.tropicos.org/Project/CE |
|  | Ehmke, A., & Eilert, U. (1993). *Solanum dulcamara* L.(Bittersweet): Accumulation of steroidal alkaloids in the plant and in different in vitro systems. In Medicinal and Aromatic Plants IV (pp. 339-352). Springer, Berlin, Heidelberg. |
|  | eMonocot database (2019). http://emonocot.org/ |
|  | Espinosa, F. J. y J. Sarukhán, 1997. Manual de Malezas del Valle de México. Claves, descripciones e ilustraciones. Universidad Nacional Autónoma de México. Fondo de Cultura Económica. México, D. F. |
|  | Euro+Med (2006-). Euro+Med PlantBase - the information resource for Euro-Mediterranean plant diversity. Published on the Internet http://ww2.bgbm.org/EuroPlusMed/ |
|  | Flora & Fauna of Libera (2019) http://www.liberianfaunaflora.org/plant-atlas-of-liberia |
|  | Flora Croatica (2019) Department of Botany , Faculty of science , FER-ZPR , University of Zagreb. http://hirc.botanic.hr/fcd/InvazivneVrste/Search.aspx |
|  | Flora de Nicaragua (2019). Catalogue of the Vascular Plants of Nicaragua. Missouri Botanical Garden, St. Louis, U.S.A. http://www.tropicos.org/Name/8500796?projectid=7 |
|  | Flora del Noroeste dé México. 2019. http//:www.herbanwmex.net/portal/index.php. |
|  | Flora do Brasil (2019) under construction. Jardim Botânico do Rio de Janeiro. Available at: http://floradobrasil.jbrj.gov.br/. |
|  | Flora of North America Editorial Committee, eds.  (1993-).  Flora of North America North of Mexico.  20+ vols.  New York and Oxford. |
|  | Flora of North America Editorial Committee, eds. (1993-). Flora of North America North of Mexico. 19+ vols. New York and Oxford. http://www.efloras.org/flora_page.aspx?flora_id=1 |
|  | Flore La Réunion (2019) http://www.mi-aime-a-ou.com/flore_ile_reunion.php |
|  | Flowers of India (2019). http://www.flowersofindia.net |
|  | Follak, S., Schleicher, C., Schwarz, M., & Essl, F. (2017). Major emerging alien plants in Austrian crop fields. Weed research, 57(6), 406-416. |
|  | Friends of Te Henui Database (2019). www.terrain.net.nz/friends-of-te-henui/ |
|  | GISIN (2019). Global Invasive Species Information Network, providing free and open access to invasive species data. USA. http://www.gisin.org. |
|  | Global Invasive Species Database (2019) http://www.iucngisd.org/gisd/ |
|  | González, A., Tezara, W., Rengifo, E., & Herrera, A. (2012). Ecophysiological responses to drought and salinity in the cosmopolitan invader Nicotiana glauca. Brazilian Journal of Plant Physiology, 24(3), 213-222. |
|  | Grubben, G. J. (2008). Plant Resources of Tropical Africa (PROTA) (Vol. 1). Prota. Online Version at https://www.prota4u.org/database/ |
|  | Hawaiian Ecosystems at Risk project (2019). Compendium of Weeds Database. http://www.hear.org/gcw/ |
|  | Hosking, J. R., Conn, B. J., Lepschi, B. J., & Barker, C. H. (2011). Plant species first recognised as naturalised or naturalising for New South Wales in 2004 and 2005. Cunninghamia, 12(1), 85-114. |
|  | Howard Morgan, V., 2019, Glossostigma cleistanthum W.R. Barker: U.S. Geological Survey, Nonindigenous Aquatic Species Database, Gainesville, FL, https://nas.er.usgs.gov/queries/FactSheet.aspx?speciesID=257, |
|  | Howell, C. J., & Sawyer, J. W. D. (2006). New Zealand naturalised vascular plant checklist (pp. 1-60). Wellington: New Zealand Plant Conservation Network. |
|  | Instituto Nacional de Tecnologia Agropecuarila (2019) Consulta atlas de Malezas http://rian.inta.gov.ar/atlasmalezas/atlasmalezasportal/default.aspx |
|  | Invasive Species South Africa (2019). Invasive Species South Africa database. http://www.invasives.org.za/ |
|  | IUCN (2019). The IUCN Red List of Threatened Species. Version 2019-1. http://www.iucnredlist.org. |
|  | Jang, J., Park, S. H., Jung, S. Y., Chang, K. S., Yang, J. C., Oh, S. H., ... & Yun, S. M. (2013). Two Newly Naturalized Plants in Korea: Senecio inaequidens DC. and S. scandens Buch.-Ham. ex D. Don. Journal of Asia-Pacific Biodiversity, 6(4), 449-453. |
|  | Jørgensen, P. M., M. H. Nee & S. G. Beck. (eds.) (2014). Catalogue of the Vascular Plants of Bolivia, Monogr. Syst. Bot. Missouri Bot. Gard. 127(1–2): i–viii, 1–1744. Missouri Botanical Garden Press, St. Louis. |
|  | Lampinen, R. & Lahti, T. 2018: Kasviatlas (2017). Helsingin Yliopisto, Luonnontieteellinen keskusmuseo, Helsinki. Levinneisyyskartat osoitteessa http://koivu.luomus.fi |
|  | Liška, J., & Soldán, Z. (2004). Alien vascular plants recorded from the Barentsburg and Pyramiden settlements, Svalbard. Preslia, 76(3), 279-290. |
|  | Lizard Island Field Guide (2010). Australian Museum's Lizard Island Research Station. http://lifg.australianmuseum.net.au/Help.html |
|  | Lockton, A.J. & Hughes, I. (2019). Species account: Berberis vulgaris. Botanical Society of the British Isles, www.bsbi.org.uk. |
|  | Lusweti A, Wabuyele E, Ssegawa P. & Mauremootoo JR (May 2011) Invasive plants of East Africa (Kenya, Uganda and Tanzania), Lucid v. 3.5 key and fact sheets. National Museums of Kenya, Makerere University, BioNET-EAFRINET, CABI & The University of Queensland (September 2011) keys.lucidcentral.org/keys/v3/EAFRINET. |
|  | Madagascar Catalogue (2019). Catalogue of the Vascular Plants of Madagascar. Missouri Botanical Garden, St. Louis, U.S.A. & Antananarivo, Madagascar http://www.tropicos.org/Project/Madagasca. |
|  | Mironga, J. M. (2014). Lessons for effective management of water hyacinth (Eichhornia crassipes (Mart.) Solms) in Kenya. *International Journal of Humanities and Social Science*, *4*(9 (1)), 118-126. |
|  | National Institute for Agro-Environmental Sciences (2007-). Asian-Pacific Alien Species Database http://www.naro.affrc.go.jp/archive/niaes/techdoc/apasd/plant.html |
|  | National Institute for Environmental Studies (2019). Invasive Species of Japan. https://www.nies.go.jp/biodiversity/invasive/index_en.html |
|  | National Tropical Botanical Garden (2019) Meet The Plants Database. https://ntbg.org/database/plants |
|  | naturalista database (2019) https://www.naturalista.mx/taxa/47126-Plantae |
|  | Navie, S., & Adkins, S. (2008). Environmental weeds of Australia: an interactive identification and information resource for over 1000 invasive plants. CRC for Australian Weed Management. https://keyserver.lucidcentral.org/weeds/data/media/Html/search.html?zoom_query= |
|  | New Crop Resource Online Program (2019) Purdue University https://hort.purdue.edu/newcrop/default.html |
|  | Online Atlas of British and Irish Flora (2019) Botanical Society of Britain & Ireland and the Biological Records Centre http://www.brc.ac.uk/plantatlas/ |
|  | Orwa, C. (2009). Agroforestree Database: A tree reference and selection guide, version 4.0. http://www. worldagroforestry. org/sites/treedbs/treedatabases. asp. |
|  | Panama Checklist (2019). Catalogue of the Vascular Plants of Panama. Missouri Botanical Garden, St. Louis, U.S.A. http://www.tropicos.org/NameSearch.aspx?projectid=4 |
|  | Parr, C. S., Wilson, M. N., Leary, M. P., Schulz, K. S., Lans, M. K., Walley, M. L., ... & Holmes, J. T. (2014). The encyclopedia of life v2: providing global access to knowledge about life on earth. Biodiversity Data Journal, (2). |
|  | Peru Checklist (2019). Catalogue of the Flowering Plants and Gymnosperms of Peru. Missouri Botanical Garden, St. Louis, U.S.A. http://www.tropicos.org/NameSearch.aspx?projectid=5 |
|  | Plants of Taiwan (2019). http://tai2.ntu.edu.tw |
|  | POWO (2019). "Plants of the World Online. Facilitated by the Royal Botanic Gardens, Kew. Published on the Internet; http://www.plantsoftheworldonline.org/ |
|  | Preston, C.D., Pearman, D. A. & Dines, T. D. 2002. New atlas of the British and Irish flora. An atlas of the vascular plants of Britain, Ireland, the Isle of Man and the Channel Islands. Oxford University Press. |
|  | Prof. Summer's Web Garden (2019) Japanese Wild Flowers http://www7a.biglobe.ne.jp/~flower_world/ |
|  | Randall, R. P. (2017). A global compendium of weeds (No. Ed. 3). RP Randall. |
|  | Randall, R. P., & Randall, R. P. (2007). The introduced flora of Australia and its weed status. Adelaide: CRC for Australian Weed Management. |
|  | Reddy, C. S. (2008). Catalogue of invasive alien flora of India. *Life Science Journal*, *5*(2), 84-89. |
|  | Reynolds, S. C. (2002). A catalogue of alien plants in Ireland. *A catalogue of alien plants in Ireland.* |
|  | Riffle, Robert Lee, Paul Craft, and Scott Zona. The encyclopedia of cultivated palms. No. Ed. 2. Timber Press, 2012. |
|  | Roos, M. C., Berendsohn, W. G., Dessein, S., Hamann, T., Hoffmann, N., Hovenkamp, P., ... & Smets, E. (2011). e-Flora Malesiana: state of the art and perspectives. *Gard. Bull. Singapore*, *63*, 189-195. http://portal.cybertaxonomy.org/flora-malesiana/ |
|  | Roskov Y., Ower G., Orrell T., Nicolson D., Bailly N., Kirk P.M., Bourgoin T., DeWalt R.E., Decock W., Nieukerken E. van, Zarucchi J., Penev L., eds. (2019). Species 2000 & ITIS Catalogue of Life, 2019 Annual Checklist. Digital resource at www.catalogueoflife.org/annual-checklist/2019. Species 2000: Naturalis, Leiden, the Netherlands. ISSN 2405-884X. |
|  | Schiemer, E. L. (1940). The Growing of Populus deltoides in South Africa. Journal of the South African Forestry Association, 5(1), 17-28. |
|  | Simpson, N.D. 1960 A Bibliographical Index of the British Flora. Privately printed, Bournemouth. Digitized and expanded by T.C.G. Rich, 2004. |
|  | South African National Biodiversity Institute (2019) PlantZ Africa http://pza.sanbi.org/ |
|  | Tela Botanica, le réseau des botanistes francophones (2019) https://www.tela-botanica.org/ |
|  | The University of Georgia - Center for Invasive Species and Ecosystem Health (2019) Invasive Plant Atlas of New England https://www.eddmaps.org/ipane/ |
|  | Tsuyuzako, S (2019). Plant Glossary, Hokkaido University http://hosho.ees.hokudai.ac.jp/~tsuyu/top/plt/a_content.html |
|  | US Forest Service (2019). Pacific Island Ecosystems at Risk (PIER). Online resource at http://www.hear.org/pier/ |
|  | USDA, NRCS. 2006. The PLANTS Database. National Plant Data Center, Baton Rouge, LA 70874-4490 USA. http://plants.usda.gov |
|  | Verloove F. (2019) Manual of the Alien Plants of Belgium. Botanic Garden Meise, Belgium. Http://alienplantsbelgium.be |
|  | Villaseñor R., J. L. y F. J. Espinosa G., 1998. Catálogo de malezas de México. Universidad Nacional Autónoma de México. Consejo Nacional Consultivo Fitosanitario. Fondo de Cultura Económica. México, D.F. |
|  | Walsh, N. G., & Stajsic, V. (2007). A census of the vascular plants of Victoria. Royal Botanic Gardens Melbourne. https://vicflora.rbg.vic.gov.au/flora/search |
|  | Wiersema J H (2019). GRIN Taxonomy. US National Plant Germplasm System. https://npgsweb.ars-grin.gov/gringlobal/search.aspx? |
|  | Wilde planten in Nederland en België (2019) https://wilde-planten.nl/namen/links.htm |
|  | Wojtyniak, K., Szymański, M., & Matławska, I. (2013). *Leonurus cardiaca* L.(motherwort): a review of its phytochemistry and pharmacology. *Phytotherapy Research*, *27*(8), 1115-1120. |
|  | Wu, S. H., Hsieh, C. F., & Rejmánek, M. (2004). Catalogue of the naturalized flora of Taiwan. Taiwania, 49(1), 16-31. |
|  | Zenni, R. D., & Ziller, S. R. (2011). An overview of invasive plants in Brazil. *Brazilian Journal of Botany*, *34*(3), 431-446. |
|  | Zipcode Zoo (2019) https://zipcodezoo.com/ |
|  | Zuloaga, F.O.; Belgrano, M.J. & Anton, A.M. (eds.) (2014). Asteraceae, Anthemideae-Gnaphalieae. Flora Argentina., Estudio Sigma Buenos Aires. Vol. 7, pars 1, pp. 1-546. http://buscador.floraargentina.edu.ar/ |
| Birds | Babbington, J (2019) Birds of Saudi Arabia. https://www.birdsofsaudiarabia.com/ |
|  | Biodiversity of Singapore (2019). Available at https://singapore.biodiversity.online/ |
|  | British Ornithologists' Club (2017-) Bulletin of the British Ornithologists' Club. Available at https://bioone.org/journals/bulletin-of-the-british-ornithologists-club/issues/2019 |
|  | CABI (2019). Invasive Species Compendium. Wallingford, UK: CAB International. www.cabi.org/isc. |
|  | Crespo-Pérez, V., Pinto, C. M., Carrión, J. M., Jarrín-E, R. D., Poveda, C., & de Vries, T. (2016). The Shiny Cowbird, Molothrus bonariensis (Gmelin, 1789)(Aves: Icteridae), at 2,800 m asl in Quito, Ecuador. Biodiversity data journal, (4). |
|  | De Juana, E., & Garcia, E. (2015). The birds of the Iberian Peninsula. Bloomsbury Publishing. |
|  | del Hoyo, J., Collar, N.J., Christie, D.A., Elliott, A., Fishpool, L.D.C., Boesman, P. and Kirwan, G.M. 2016. HBW and BirdLife International Illustrated Checklist of the Birds of the World. Volume 2: Passerines. Lynx Edicions and BirdLife International, Barcelona, Spain and Cambridge, UK. |
|  | del Hoyo, J., Elliott, A., Sargatal, J., Christie, D.A. & Kirwan, G. (eds.) (2019). Handbook of the Birds of the World Alive. Lynx Edicions, Barcelona https://www.hbw.com/ |
|  | Global Invasive Species Database (2019) http://www.iucngisd.org/gisd/ |
|  | IUCN (2019). The IUCN Red List of Threatened Species. Version 2019-1. http://www.iucnredlist.org. |
|  | Lepage, D., Vaidya, G., & Guralnick, R. (2014). Avibase–a database system for managing and organizing taxonomic concepts. ZooKeys, (420), 117. https://avibase.bsc-eoc.org/avibase.jsp |
|  | Lever, C. (2005). Naturalised birds of the world. A&C Black. |
|  | Myers, P., R. Espinosa, C. S. Parr, T. Jones, G. S. Hammond, and T. A. Dewey. 2019. The Animal Diversity Web (online). Accessed at https://animaldiversity.org. |
|  | Parr, C. S., Wilson, M. N., Leary, M. P., Schulz, K. S., Lans, M. K., Walley, M. L., ... & Holmes, J. T. (2014). The encyclopedia of life v2: providing global access to knowledge about life on earth. Biodiversity Data Journal, (2). |
|  | Pyle, R.L., and P. Pyle. 2017. The Birds of the Hawaiian Islands: Occurrence, History, Distribution, and Status. B.P. Bishop Museum, Honolulu, HI, U.S.A. Version 2 http://hbs.bishopmuseum.org/birds/rlp-monograph |
|  | Shieh, B. S., Lin, C. J., & Liang, S. H. (2016). Breeding biology of the invasive Asian Glossy Starling (Aplonis panayensis) in urban parks of Kaohsiung City, southern Taiwan. Taiwan Journal of Forest Science, 31, 63-70. |
|  | Vall‐llosera, M., & Sol, D. (2009). A global risk assessment for the success of bird introductions. Journal of Applied Ecology, 46(4), 787-795. |
| Mammals | IUCN (2019). The IUCN Red List of Threatened Species. Version 2019-1. http://www.iucnredlist.org. |
