# Supplementary material for: Global terrestrial invasions: Where naturalised birds, mammals, and plants might spread next and what affects this process
Source: PLoS Biol. 2023 Nov 14;21(11):e3002361. doi: 10.1371/journal.pbio.3002361 (PMC10645288; doi:10.1371/journal.pbio.3002361)
Supplement: S7 Table — Taxa indicate which taxonomic group the source covered. All sources accessed March 2018. (DOCX) [file pbio.3002361.s008.docx]

**Table S7:** List of sources that provided either first introduction date or first observed record of introduced species. Taxa indicates which taxonomic group the source covered. All sources accessed March 2018.

| Citation | Taxa |
| --- | --- |
| Atlas of Living Australia website (2019) at http://www.ala.org.au. | Plants |
| Dyer, E. E., Redding, D. W., & Blackburn, T. M. (2017). The global avian invasions atlas, a database of alien bird distributions worldwide. Scientific data, 4, 170041. | Birds |
| Early, R., & Sax, D. F. (2014). Climatic niche shifts between species' native and naturalized ranges raise concern for ecological forecasts during invasions and climate change. Global ecology and biogeography, 23(12), 1356-1365. | Plants |
| European Commission - Joint Research Centre (2019). European Alien Species Information Network (EASIN) https://easin.jrc.ec.europa.eu/ | Plants |
| Long, J. (2003). Introduced Mammals of the World (CSIRO Publishing: Melbourne). | Mammals |
| Seebens, H., Blackburn, T. M., Dyer, E. E., Genovesi, P., Hulme, P. E., Jeschke, J. M., ... & Bacher, S. (2017). No saturation in the accumulation of alien species worldwide. Nature communications, 8, 14435. | Plants/Birds/Mammals |
