# Supplementary material for: Global terrestrial invasions: Where naturalised birds, mammals, and plants might spread next and what affects this process
Source: PLoS Biol. 2023 Nov 14;21(11):e3002361. doi: 10.1371/journal.pbio.3002361 (PMC10645288; doi:10.1371/journal.pbio.3002361)
Supplement: S8 Table — All parameters in the “all variables trialled” list were included individually in a univariate Bayesian hierarchical model with region as the hierarchical effect. Parameters in bold in this column are variables that were significant (based on 90% CIs) either globally or in some at least some realms in the univariate model, and which were trialled in the multivariate model. Parameters in the “final variable list” are the parameters retained in the model for which results are described in the main manuscript. (DOCX) [file pbio.3002361.s009.docx]

**Table S8:** A summary of parameters trialled for plants, birds and mammals. All parameters in the “all variables trialled” list were included individually in a univariate Bayesian hierarchical model with region as the hierarchical effect. Parameters in bold in this column are variables that were significant (based on 90% CIs) either globally or in some at least some realms in the univariate model, and which were trialled in the multivariate model. Parameters in the “final variable list” are the parameters retained in the model for which results are described in the main manuscript.

| Taxonomic Group | All variables trialled | Final variable list |
| --- | --- | --- |
| Plants | Categorical: Invasive status, **realm**, growth form, aquatic status, horticultural status  Continuous: **local recording effort,** time since introduction, **height (logged), dispersal category**, habitat diversity, average seed number (logged), **days till flowering (logged),** **habitat fragmentation (contagion), habitat fragmentation (clumpiness)** | **Time** **since introduction**  **Days till flowering (logged)**  **Local recording effort**  **Realm** |
| Birds | Categorical: Invasive status, **realm**, diet category  Continuous: local recording effort, **time since introduction**, average body weight (logged), average wingspan (logged), **natal dispersal distance (logged)**, clutch size, clutches per year, habitat diversity, **habitat fragmentation (contagion),** habitat fragmentation (clumpiness) | **Natal dispersal distance (logged)**  **Time since introduction**  **Habitat Fragmentation (contagion)**  **Realm** |
| Mammals | Categorical: Invasive status, **realm**  Continuous: local recording effort, **time since introduction**, **average body mass (logged), home range size (logged), natal dispersal distance (logged),** habitat diversity, interbirth interval, litter size, age of sexual maturity, brain residual size, **habitat fragmentation (contagion),** habitat fragmentation (clumpiness) | **Natal dispersal distance (logged)**  **Habitat Fragmentation (contagion)**  **Realm** |
