# Supplementary material for: Global terrestrial invasions: Where naturalised birds, mammals, and plants might spread next and what affects this process
Source: PLoS Biol. 2023 Nov 14;21(11):e3002361. doi: 10.1371/journal.pbio.3002361 (PMC10645288; doi:10.1371/journal.pbio.3002361)
Supplement: S9 Table — This model contains 1 continuous parameter (beta1) and 1 hierarchical effect (cat1). N is the total sample size, N_cat1 is the total number of levels of cat1, y is the response variable, x1 is the continuous predictor variable (scaled to have a mean of 0 and a standard deviation of 2). (DOCX) [file pbio.3002361.s010.docx]

**Table S9:** An example Bayesian model based on a beta distribution with a logit link written in JAGs. This model contains one continuous parameter (beta1), and one hierarchical effect (cat1). N is the total sample size, N_cat1 is the total number of levels of cat1, y is the response variable, x1 is the continuous predictor variable (scaled to have a mean of 0 and a standard deviation of 2).

| model  {  *### MODEL section*  for (t in 1:N) {  *#define a beta distribution*  y[t] ~ dbeta(a[t], b[t])  *#create a parameter for a post-predictive check*  y_pred[t] ~ dbeta(a[t], b[t])  *#extract loglikelihood (needed for WAIC)*  LogLik[t] <- log(dbeta(y[t], a[t], b[t]))    *#define a and b terms for beta distribution*  a[t] <- mu[t] * phi  b[t] <- (1 - mu[t]) * phi  *#define logit link. Equation is a simple linear a+bx with a hierarchical effect on b*  logit(mu[t]) <- alpha[cat1[t]] +  beta1[cat1[t]] * x1[t]  }    *### PRIOR section*  *# Priors will loop across all levels of hierarchical effect.*  for(j in 1:N_cat1){  alpha[j] ~ dnorm(mu_alpha, sigma_alpha^-2)  beta1[j] ~ dnorm(mu_beta1, sigma_beta1^-2)  }  *# Priors for continuous parameter. Intercept varies around -1, continuous parameter varies around 0*  mu_alpha ~ dnorm(-1, 2^-2)  mu_beta1 ~ dnorm(0, 1^-2)  *#specify hierarchical priors as a half-Cauchy distribution with a long tail.*  sigma_alpha ~ dt(0,5,1)T(0,)  sigma_beta1 ~ dt(0,5,1)T(0,)    *#define prior for dispersion of the model*  phi ~ dunif(0, 10)  } |
| --- |
