# Supplementary material for: Global terrestrial invasions: Where naturalised birds, mammals, and plants might spread next and what affects this process
Source: PLoS Biol. 2023 Nov 14;21(11):e3002361. doi: 10.1371/journal.pbio.3002361 (PMC10645288; doi:10.1371/journal.pbio.3002361)
Supplement: S10 Table — Models 1 and 2 incorporate all variables that were important either at a global level or in some realms in univariate models (based on 90% CIs). Models were subsequently created as viable alternatives either to assist model convergence or to trial dropping unimportant variables (i.e., posterior estimates centred at or near 0). The final model used in the main manuscript is presented in bold. Alternative models that are equally viable are in italics. (DOCX) [file pbio.3002361.s011.docx]

**Table S10:** A summary of all Bayesian hierarchical models trialled to correlate range filling with various traits and spatial features for plants. Models 1 and 2 incorporate all variables that were important either at a global level or in some realms in univariate models (based on 90% CIs). Models were subsequently created as viable alternatives either to assist model convergence or to trial dropping unimportant variables (i.e. posterior estimates centred at or near 0). The final model used in the main manuscript is presented in bold. Alternative models which are equally viable are in italics.

| model | equation | Sample Size | Convergence problems? | pD | DIC | Pseudo R-Squared | RMSE | Issues with LOO? | Notes |
| --- | --- | --- | --- | --- | --- | --- | --- | --- | --- |
| 1 | Range filling ~ Years since introduction + Days till flowering (logged) + Dispersal Category + Local recording effort + 1\|Region | 484 | Yes | 21.33 | -1065.27 | 0.31 | 2.23 | No | All variables significant in univariate models, but covariance between dispersal and height was too great for both to be included in the same model |
| *2* | *Range filling ~ Years since introduction + Days till flowering (logged) + Height (logged) + Local recording effort + 1\|Region* | *484* | *No* | *23.16* | *-1061.78* | *0.32* | *2.22* | *No* |  |
| 3 | **Range filling ~ Years since introduction + Days till flowering (logged) + Local recording effort + 1\|Region** | **484** | **No** | **18.96** | **-1065.79** | **0.31** | **2.22** | **No** | **Height and dispersal were trialled for removal as they had least influence in model 2** |
| 4 | Range filling ~ Days till flowering (logged) + Local recording effort + 1\|Region | 484 | Yes | 18.36 | -1012.53 | 0.22 | 2.16 | No | Years since introduction was trialled for removal as it had least influence in model 3 |
