# Supplementary material for: Global terrestrial invasions: Where naturalised birds, mammals, and plants might spread next and what affects this process
Source: PLoS Biol. 2023 Nov 14;21(11):e3002361. doi: 10.1371/journal.pbio.3002361 (PMC10645288; doi:10.1371/journal.pbio.3002361)
Supplement: S12 Table — Model 1 incorporates all variables that were important either at a global level or in some realms in univariate models (based on 90% CIs). Models were subsequently created as viable alternatives either to assist model convergence or to trial dropping unimportant variables. The final model used in the main manuscript is presented in bold. (DOCX) [file pbio.3002361.s013.docx]

**Table S12:** A summary of all Bayesian hierarchical models trialled to correlate range filling with various traits and spatial features for mammals. Models 1 is incorporates all variables that were important either at a global level or in some realms in univariate models (based on 90% CIs). Models were subsequently created as viable alternatives either to assist model convergence or to trial dropping unimportant variables. The final model used in the main manuscript is presented in bold.

| model | equation | Sample Size | Convergence problems? | pD | DIC | Pseudo R-Squared | RMSE | Issues with LOO? | Notes |
| --- | --- | --- | --- | --- | --- | --- | --- | --- | --- |
| 1 | Range filling ~ Years since Introduction + Natal Dispersal (logged km) + Habitat Fragmentation (contagion) + Region | 46 | Yes | 23.17 | -86.73 | 0.50 | 2.99 | Yes |  |
| 2 | **Range filling ~ Natal Dispersal (logged km) + Habitat Fragmentation (contagion) + 1\|Region** | **46** | **No** | **22.20** | **-87.95** | **0.50** | **2.98** | **No** | Years since introduction was trialled for removal as it had least influence in models 1 |
| 3 | Range filling ~ Years since Introduction + Habitat Fragmentation (contagion) + 1\|Region | 39 | No | 26.80 | -80.74 | 0.56 | 2.85 | No | Natal dispersal was trialled for removal as it had least influence in models 2. |
| 4 | Range filling ~ Habitat Fragmentation (contagion) + 1\|Region | 51 | No | 25.26 | -102.87 | 0.42 | 2.96 | Yes | Most parsimonious model also trialled due to smaller sample size. |
