# Supplementary material for: Global terrestrial invasions: Where naturalised birds, mammals, and plants might spread next and what affects this process
Source: PLoS Biol. 2023 Nov 14;21(11):e3002361. doi: 10.1371/journal.pbio.3002361 (PMC10645288; doi:10.1371/journal.pbio.3002361)
Supplement: S13 Table — Model numbers correspond to those in S10 Table. Estimates for parameters retained in the final model are given as the mean estimate of all posterior draws, with the 5% and 95% estimates as confidence intervals in parentheses. Parameter estimates are given as the linear slope of the logit link equation. When parameter estimates vary across realms, this is indicated by providing the names of the realms in which it varies (Aus = Australian, Nea = Nearctic, Neo = Neotropical). Model verification data are given for the final models, including sample size, DIC of the model, the effective number of parameters (pD), and correlation of the linear predictor against the link transformed response given as a pseudo R-squared. Note that a negative effect of recording effort means that more recorder effort in the potential naturalised range corresponded to lower range filling. Model 2 is presented in the main manuscript. (DOCX) [file pbio.3002361.s014.docx]

**Table S13:** Correlates of range filling for the two best, equally plausible models for plants. Model numbers correspond to those in table S10. Estimates for parameters retained in the final model are given as the mean estimate of all posterior draws, with the 5% and 95% estimates as confidence intervals in parentheses. Parameter estimates are given as the linear slope of the logit link equation. When parameter estimates vary across realms, this is indicated by providing the names of the realms in which it varies (Aus = Australian, Nea = Nearctic, Neo=Neotropical). Model verification data are given for the final models, including sample size, Deviance Information Criterion (DIC) of the model, the effective number of parameters (pD), and correlation of the linear predictor against the link transformed response given as a pseudo R-squared. Note that a negative effect of recording effort means that more recorder effort in the potential naturalised range corresponded to lower range filling. Model 2 is presented in the main manuscript.

|  | **Model Parameter** | **Estimate** | **95% CI** | **Differences between realms?** | **Model Verification** | **Estimate** |
| --- | --- | --- | --- | --- | --- | --- |
| **Plants – Model 2** | Intercept | -2.32 | (-1.65, -3.00) | Aus | Sample Size | 484 |
|  | Years since Introduction | 0.36 | (0.55, 0.16) |  | DIC | -1062.07 |
|  | Days till Flowering (logged) | -0.11 | (0.05, -0.25) | Aus, Nea | pD | 23.09 |
|  | Local recording effort | 0.31 | (0.28, -0.90) | Aus | Pseudo R-Squared | 0.31 |
|  | Height (logged) | -0.03 | (0.16, -0.20) |  |  |  |
| **Plants – Model 3** | Intercept | -2.27 | (-1.59, -3.02) | Aus | Sample Size | 484 |
|  | Years since Introduction | 0.35 | (0.53, 0.15) |  | DIC | -1065.79 |
|  | Days till Flowering (logged) | -0.13 | (0.03, -0.26) | Aus, Nea | pD | 18.79 |
|  | Local recording effort | -0.29 | (0.34, -0.86) | Aus | Pseudo R-Squared | 0.31 |
