# Supplementary figures and images for: Global terrestrial invasions: Where naturalised birds, mammals, and plants might spread next and what affects this process
Source: PLoS Biol. 2023 Nov 14;21(11):e3002361. doi: 10.1371/journal.pbio.3002361 (PMC10645288; doi:10.1371/journal.pbio.3002361)

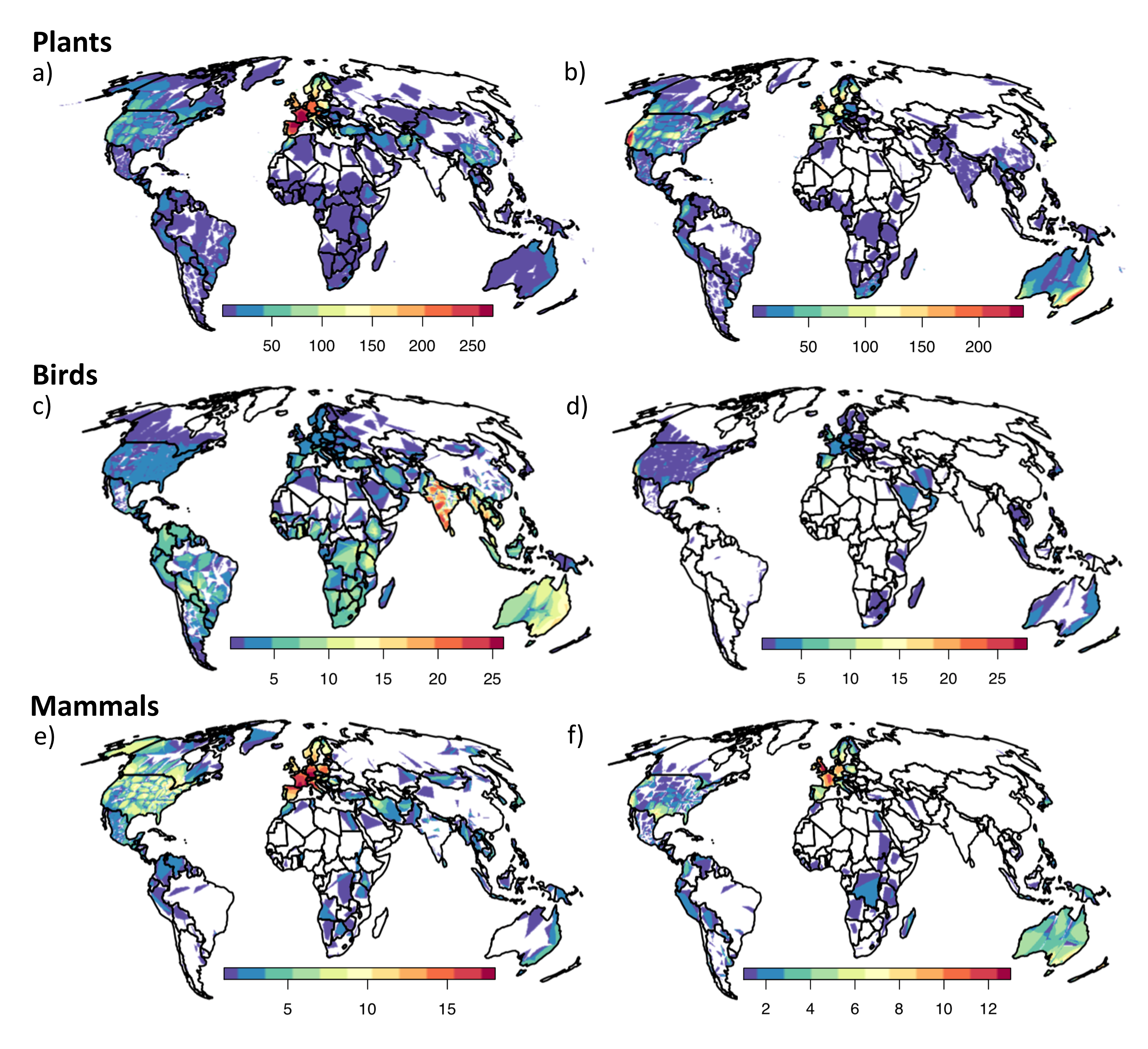

Supplement: S1 Fig — Colours represent number of species. The data underlying this figure can be found in https://doi.org/10.5281/zenodo.8205905. Country and continent outlines were produced by the International Working Group on Taxonomic Databases for Plant Sciences (TDWG), specifically the WGSRPD Level 4 boundaries; data and usage notes can be found at (https://github.com/tdwg/wgsrpd). (PNG) [file pbio.3002361.s015.png]

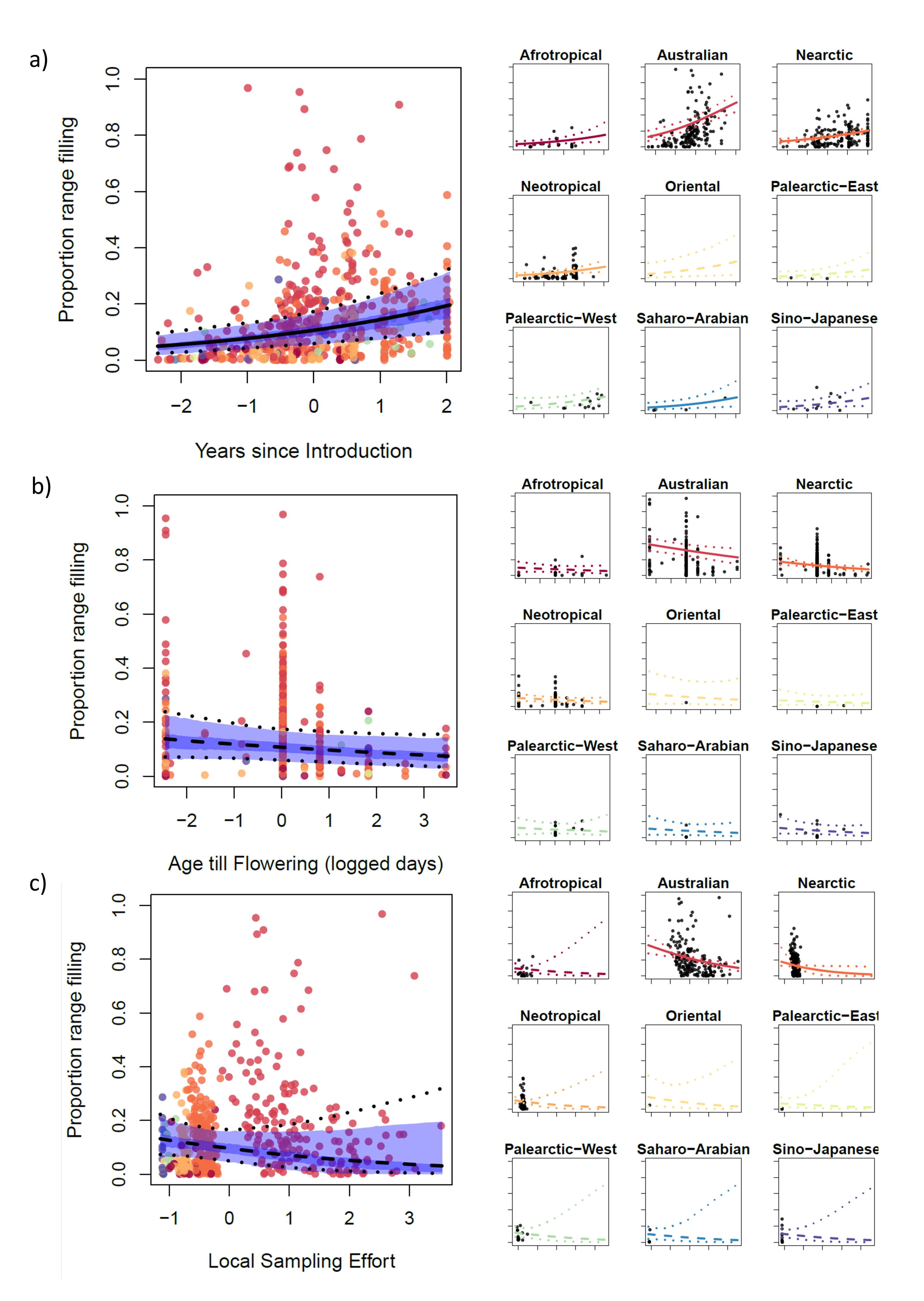

Supplement: S2 Fig — On the left are global trends for each parameter, on the right are the realm hierarchical effects. (a) Years since introduction, (b) age of first flowering event; (c) the estimated local sampling effort. A solid line signifies the estimate was consistently above or below 0 in >95% of simulations (and therefore judged as significant), a dashed line means it was not. The lighter shaded area shows the 95% probability density interval for the parameter estimate, and the darker shows the 50% interval. The data underlying this figure can be found in https://doi.org/10.5281/zenodo.8205905. (PNG) [file pbio.3002361.s016.png]

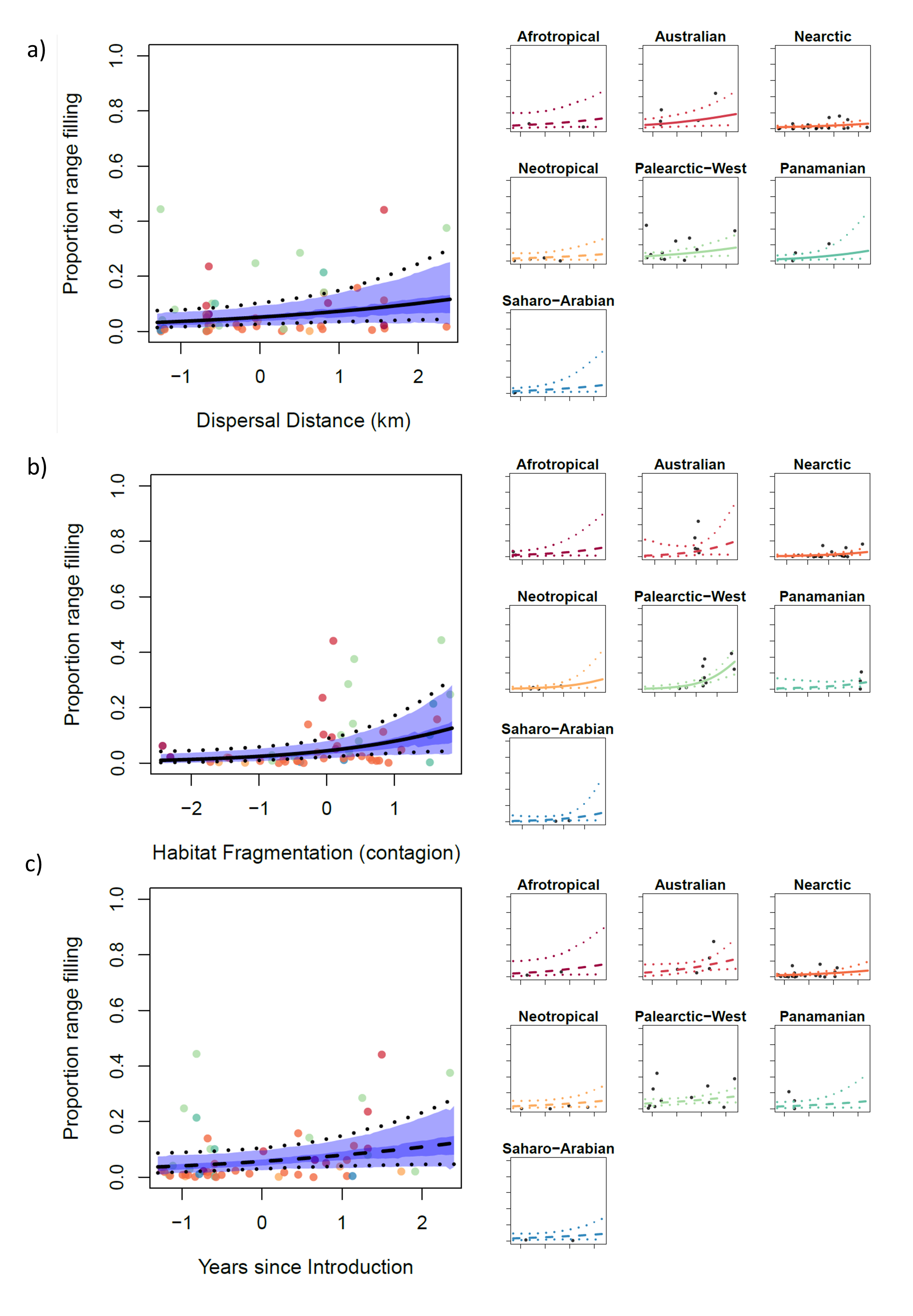

Supplement: S3 Fig — On the left are global trends for each parameter, on the right are the realm hierarchical effects. (a) Natal dispersal distance (km), (b) fragmentation of suitable climate (contagion), (c) years since introduction. A solid line signifies the estimate was consistently above or below 0 in >95% of simulations (and therefore judged as significant), a dashed line means it was not. The lighter shaded area shows the 95% probability density interval for the parameter estimate, and the darker shows the 50% interval. The data underlying this figure can be found in https://doi.org/10.5281/zenodo.8205905. (PNG) [file pbio.3002361.s017.png]

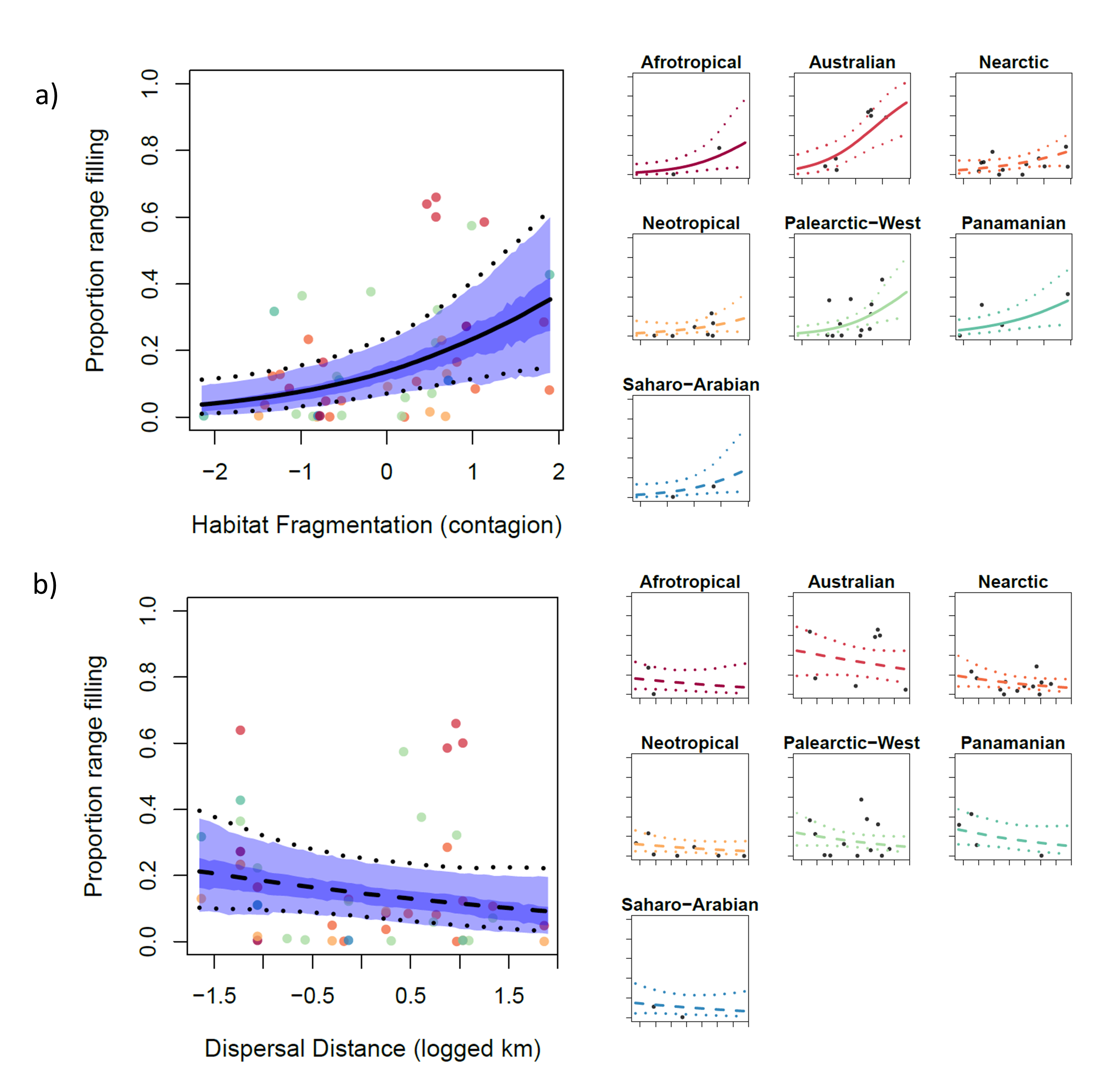

Supplement: S4 Fig — On the left are global trends for each parameter, on the right are the realm hierarchical effects. (a) Fragmentation of suitable climate (contagion), (b) dispersal distance (logged km). A solid line signifies the estimate was consistently above or below 0 in >95% of simulations (and therefore judged as significant), a dashed line means it was not. The lighter shaded area shows the 95% probability density interval for the parameter estimate, and the darker shows the 50% interval. The data underlying this figure can be found in https://doi.org/10.5281/zenodo.8205905. (PNG) [file pbio.3002361.s018.png]

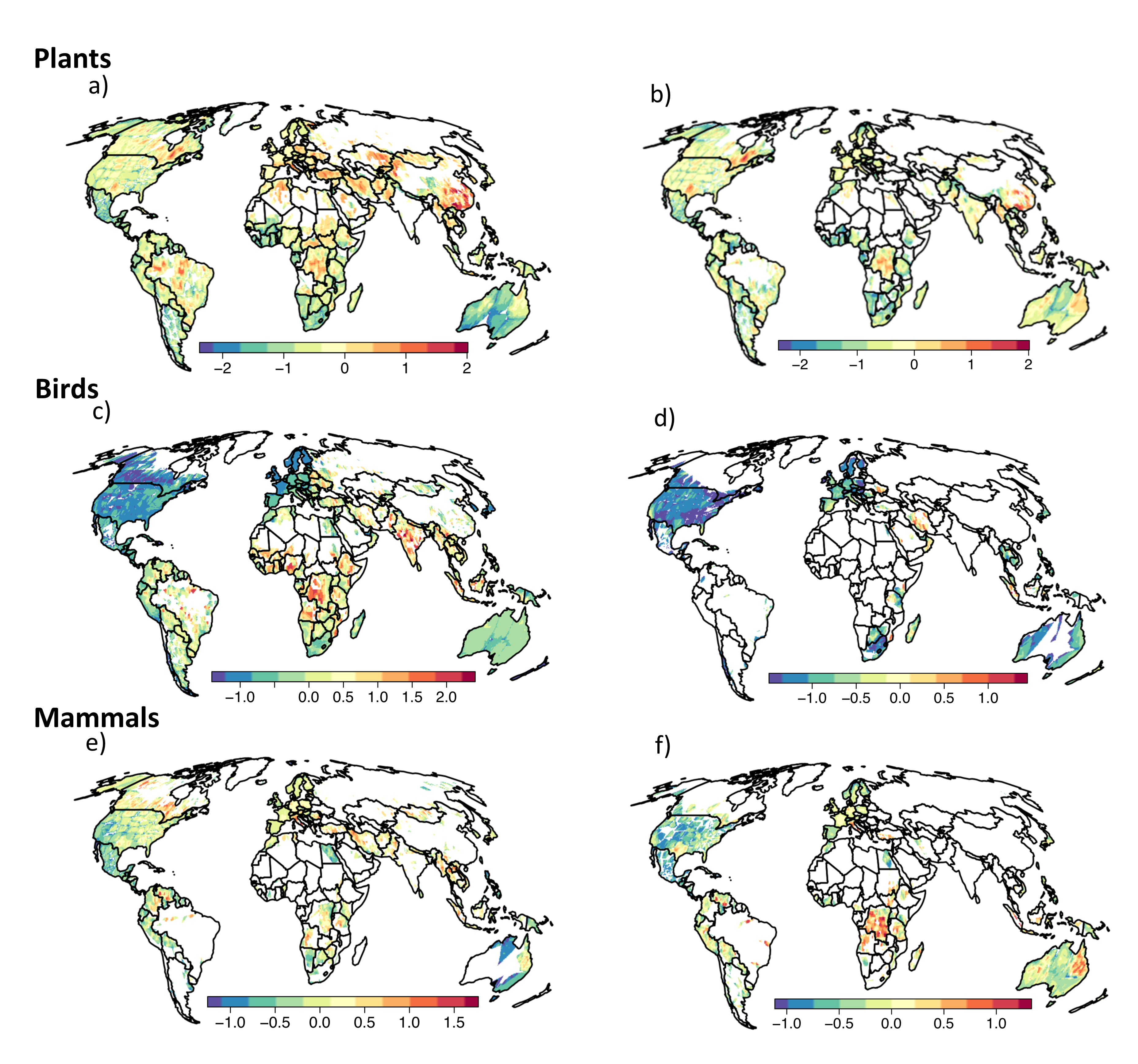

Supplement: S5 Fig — Colours represent the adjusted relative number of species. The data underlying this figure can be found in https://doi.org/10.5281/zenodo.8205905. Country and continent outlines were produced by the International Working Group on Taxonomic Databases for Plant Sciences (TDWG), specifically the WGSRPD Level 4 boundaries; data and usage notes can be found at (https://github.com/tdwg/wgsrpd). (PNG) [file pbio.3002361.s019.png]

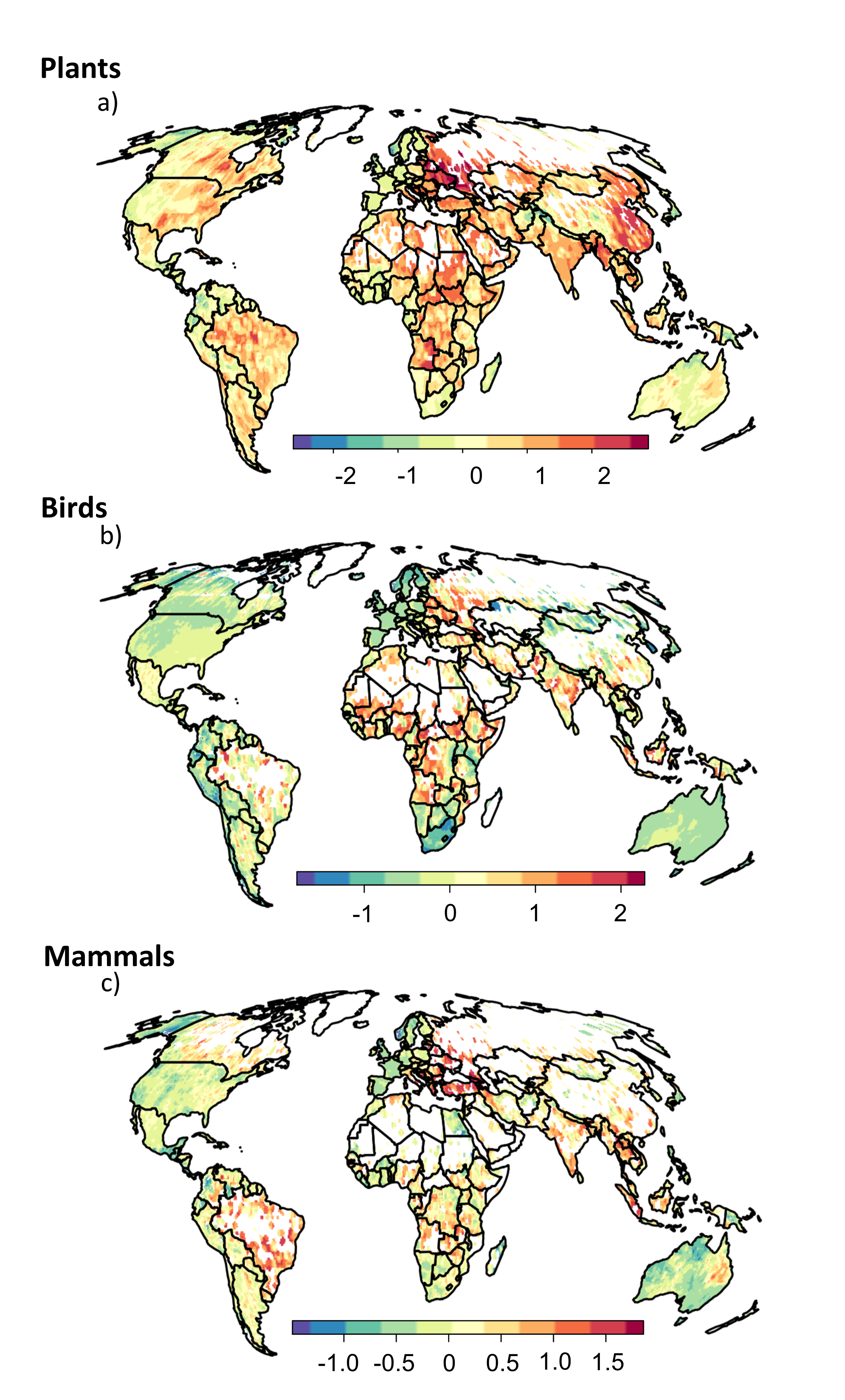

Supplement: S6 Fig — The number of species that could spread to each 10-min grid-cell is calculated in the same way as in Fig 1). This number was then multiplied by a measure of recording effort (proportion of known species per grid-cell that are actually reported in GBIF data, see Methods and Meyer and colleagues for a full description) to compensate for potential over- or under-recording of species. The data underlying this figure can be found in https://doi.org/10.5281/zenodo.8205905. Country and continent outlines were produced by the International Working Group on Taxonomic Databases for Plant Sciences (TDWG), specifically the WGSRPD Level 4 boundaries; data and usage notes can be found at (https://github.com/tdwg/wgsrpd). (PNG) [file pbio.3002361.s020.png]

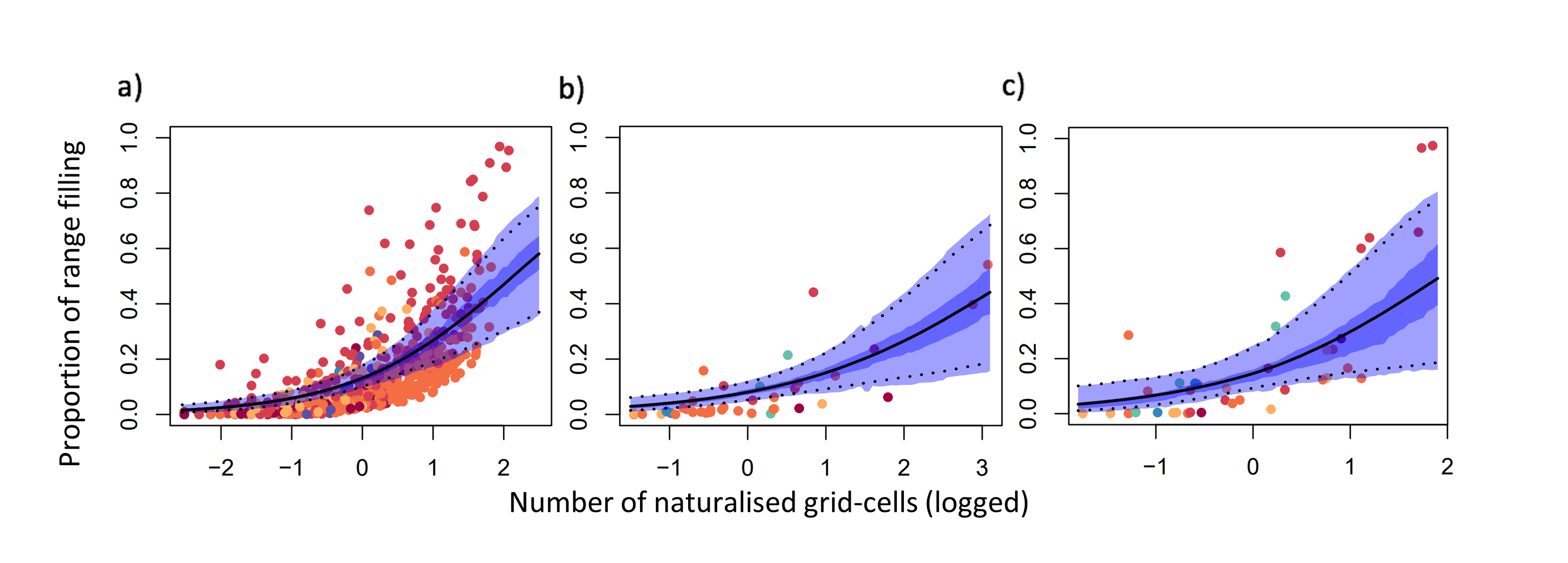

Supplement: S7 Fig — A solid line signifies the estimate was consistently above or below 0 in >95% of simulations (and therefore judged as significant), a dashed line means it was not. The lighter shaded area shows the 95% probability density interval for the parameter estimate, and the darker shows the 50% interval. Point colour represents region, but as parameter estimates did not vary between region, only the global regression line is shown. The data underlying this figure can be found in https://doi.org/10.5281/zenodo.8205905. (PNG) [file pbio.3002361.s021.png]

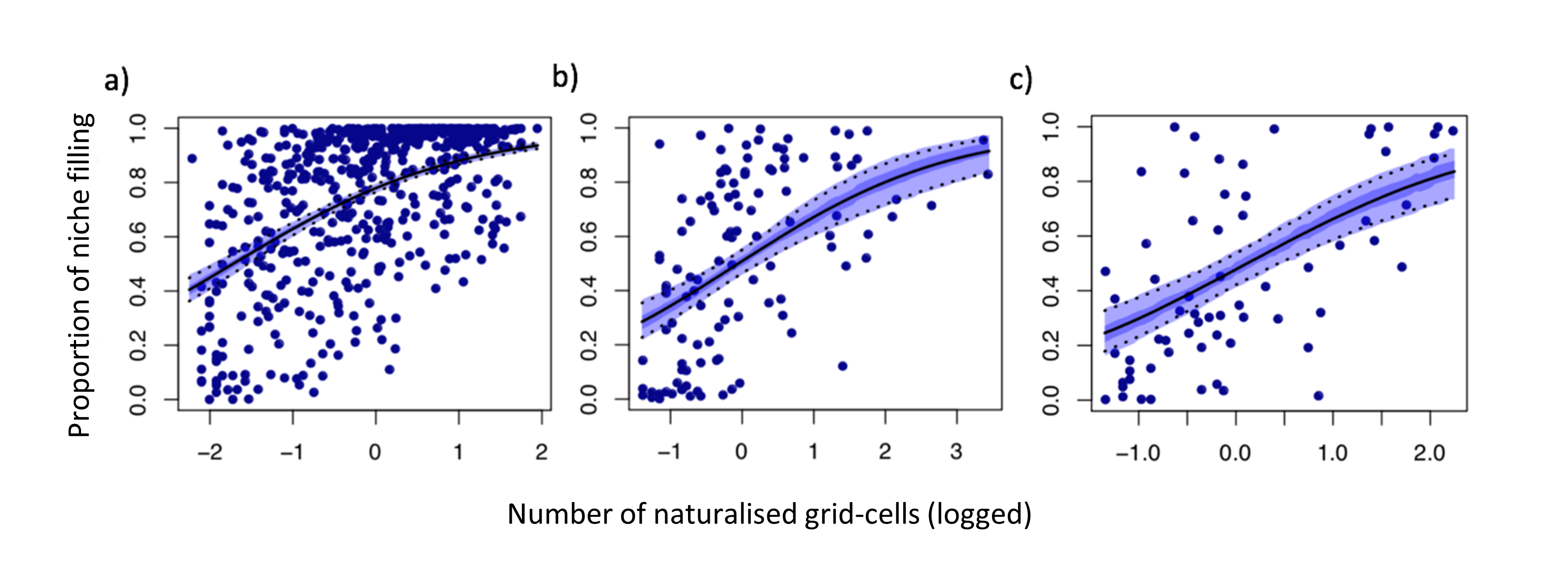

Supplement: S8 Fig — A solid line signifies the estimate was consistently above or below 0 in >95% of simulations (and therefore judged as significant), a dashed line means it was not. The lighter shaded area shows the 95% probability density interval for the parameter estimate, and the darker shows the 50% interval. The data underlying this figure can be found in https://doi.org/10.5281/zenodo.8205905. (PNG) [file pbio.3002361.s022.png]

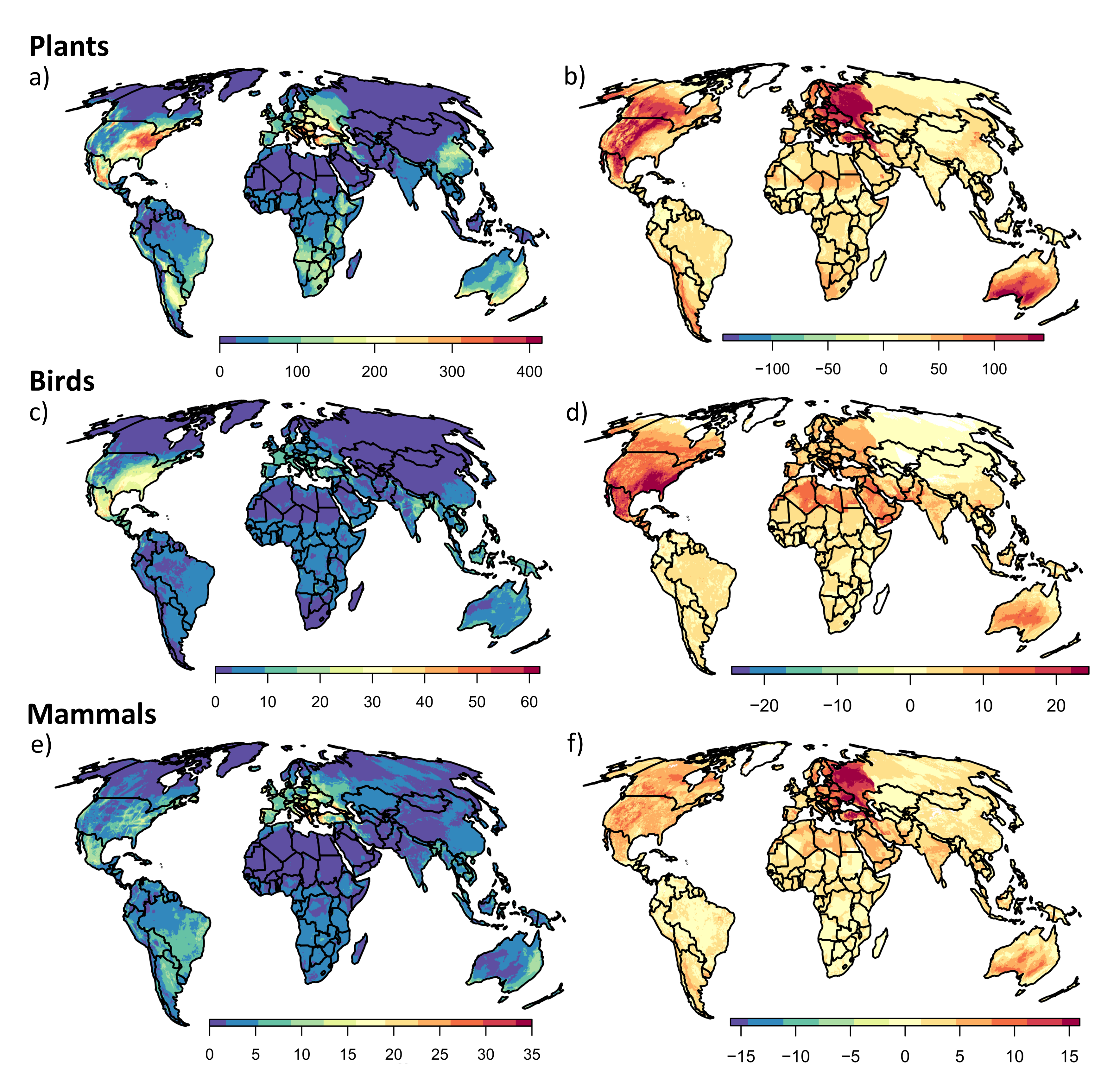

Supplement: S9 Fig — Figures on the left depict the number of species that could spread to that area and are already naturalised within that realm. Figures on the right depict the discrepancy between threat metrics for 100% (Fig 1) and 70% niche overlap maps. Positive values mean that the number of species that are calculated to spread using 100% of occupied native climate is greater than when using 70% of occupied native climate. The data underlying this figure can be found in https://doi.org/10.5281/zenodo.8205905. Country and continent outlines were produced by the International Working Group on Taxonomic Databases for Plant Sciences (TDWG), specifically the WGSRPD Level 4 boundaries; data and usage notes can be found at (https://github.com/tdwg/wgsrpd). (PNG) [file pbio.3002361.s023.png]

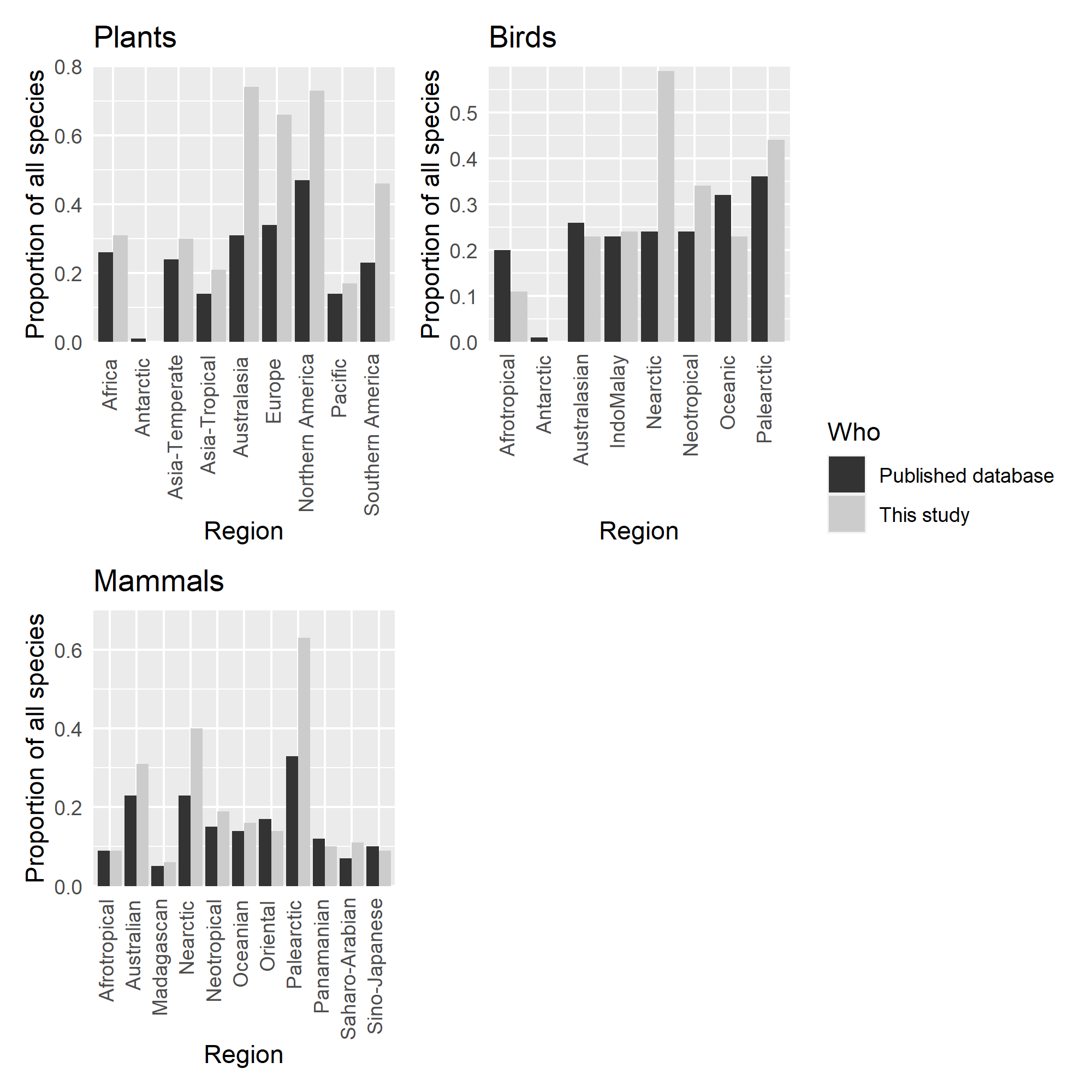

Supplement: S10 Fig — Note that the geographic regions are specific to each published database, not to our study. Also note that proportions for published databases and for our study sum to >1, as some species have naturalised in multiple regions. The data underlying this figure can be found in https://doi.org/10.5281/zenodo.8205905. (PNG) [file pbio.3002361.s024.png]

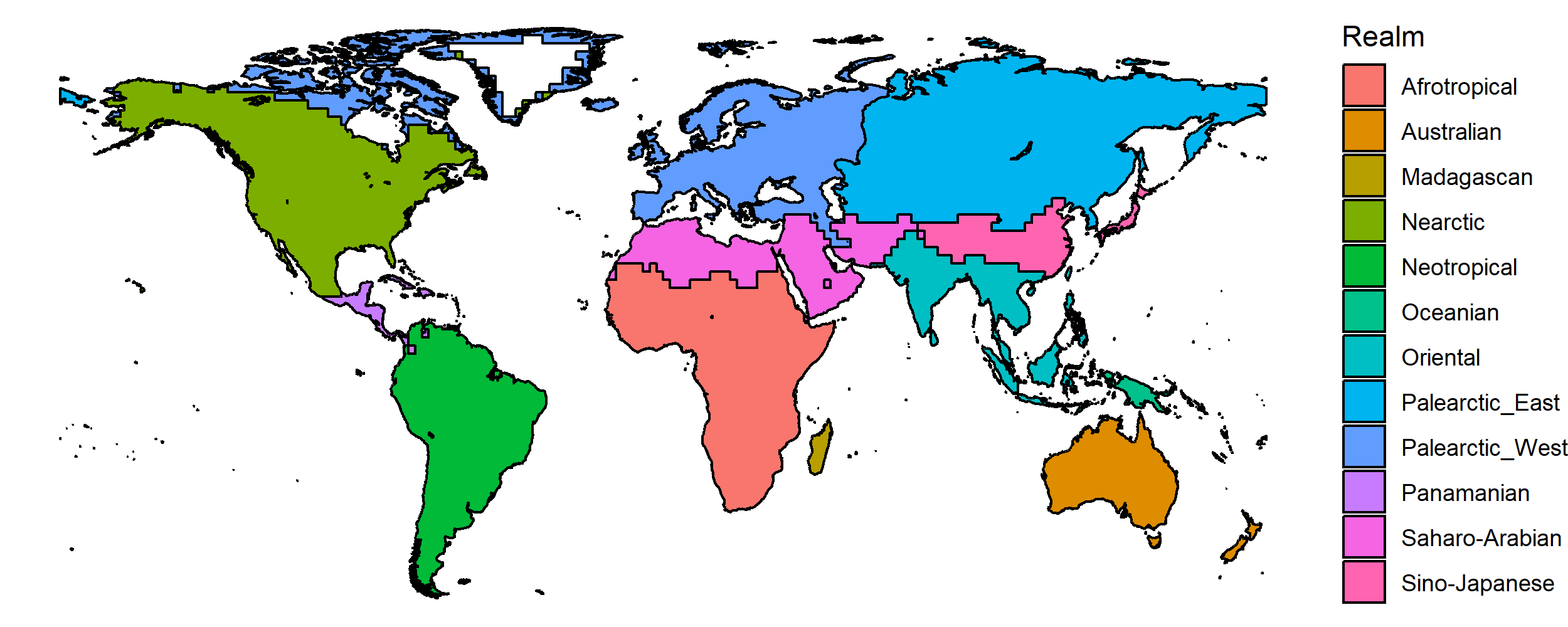

Supplement: S11 Fig — Realms were defined by Holt and colleagues, with the addition of a line between the western and eastern Palearctic along the Ural Mountains. The data for this figure is not available from the authors of this study, but can be attained from Holt and colleagues. Credit: Journal Science/AAAS. (PNG) [file pbio.3002361.s025.png]
