# Supplementary material for: Global terrestrial invasions: Where naturalised birds, mammals, and plants might spread next and what affects this process
Source: PLoS Biol. 2023 Nov 14;21(11):e3002361. doi: 10.1371/journal.pbio.3002361 (PMC10645288; doi:10.1371/journal.pbio.3002361)
Supplement: Attachment — Submitted filename: Response to Reviewers.pdf [file pbio.3002361.s026.pdf]

PLOS Biology

REVIEWERS' COMMENTS:

Reviewer #1:

This paper globally quantifies the regional risk of invasion of alien birds, mammals and plants according to their climatic suitability. They further compare the obtained risk with management capacities from a previous study and lastly investigate how their findings would change with changes in climate. These are important and novel topics and they are addressed here with adequate methods. I congratulate the authors on addressing these important issues. This is certainly an interesting study for the readers of PLoS Biology and should surely be published.

Thank you!

However, I feel that in the Introduction and Abstract the paper a little bit oversells the importance of the findings by making many deterministic statements (things "will" happen) and by taking the (climatic) suitability of a location as the only factor determining the risk or threat of colonization. Climatic suitability is necessary for invasion, but it only poses a risk if species can reach the locations. This is not really considered in the paper, although the authors do have data on the current distribution of the species and therefore could e.g. take the distance to potential source populations into account for each species or model only the suitability of regions that are reachable for each species (i.e. around source populations). These restrictions are well discussed later in the discussion of the paper, but I feel that they should at least be introduced early in the Introduction in order not to raise unrealistic expectations. They certainly should be mentioned in the abstract or the findings should be sold less boldly.

We think this is a very important comment and wanted to address it as fully as possible. We felt the reality of the threat would be best addressed by a comprehensive analysis of why so many introduced species have yet to fill all their climatically potential ranges. Having now included this analysis in the paper we feel it provides a much deeper insight into whether species could spread more widely, and a comprehensive assessment of what underlies invasion processes more generally. We investigated multiple species and landscape characteristics, but those most relevant to ‘distance to potential source populations’ are the fragmentation of suitable climate, dispersal ability, and date of introduction. Our results indicate that self-powered dispersal, and thus distance from source population, is important for birds and mammals, but not plants. This helps us interpret the threat of continued invasive spread more clearly – please see the new discussion. We are grateful to the reviewer for spurring us to do this!

I do have to confess that I am not an expert in SDM, thus I cannot comment on the technical details of the methods applied. My methodological comments address rather conceptual issues and thus should be taken with a grain of salt.

In the following, please find my detailed comments:

l. 22: "in their native and alien ranges"? Only mentioning native ranges seems a bit

misleading to me as you used climatic conditions in native and introduced areas in the analyses (at least to obtain the PCAs). As it is widely known, and you discussed this, that using only the environmental conditions in the native range to predict suitable areas can lead to biases, it might be good to be clear about this.

The reviewer is correct that we only use the climate species occupy in their native ranges to project their potential ranges in the realms where they have naturalised. We agree that climate niche expansion following naturalisation often occurs, and that this could increase species' potential naturalised ranges. However, there are multiple reasons for niche expansion, including niche evolution, restricted native ranges, and altered biotic interactions in the naturalised realm. Without knowing which factor caused expansion into a given climate for each individual species, we cannot know whether the species could occupy similar climate elsewhere in their naturalised range. Therefore, our predictions are conservative – species may be capable of naturalising further than we predict. We have clarified the method and the potential for underprediction in lines 262-266.

l. 23: "Naturalized species will spread much more widely ..." Just because areas are climatically suitable doesn't mean that they will be colonized by the species, e.g. if these areas cannot be reached by natural dispersal. Maybe this can be formulated more carefully or (even better) the vicinity of areas to current alien populations might be incorporated into the forecasts. Also, the following statements could be formulated less deterministically.

We very much agree with the reviewer and have now reformulated the text throughout the MS, in light of the additional analyses we have added.

l. 25: I am curious if the authors have indications that the general capacity of countries to mitigate impacts is a good predictor of actually mitigating colonization, spread or impacts.

We have now removed this part of the analysis.

l. 69: Are these numbers referring to all native birds, mammals and plants? Maybe clarify that the native species are the ones that are naturalized somewhere in their native range; I found this confusing and had to read the Methods first.

This is now made clear in the supplementary table (referred to in line 80).

l. 88-90: This is a key question: are the species in these adjacent areas the species for which the regions are suitable? Suitability of an area is a risk for invasion only if species for which it is suitable can actually reach these areas. I think the paper would tremendously gain if the authors could incorporate a way to relate the suitability to the distance of potential source populations. If no source population is close by, it is unlikely that even suitable regions would be colonized. Thus, the suitability alone is only part of the risk, but the authors have the data about the distribution of the species and could actually take the distance to potential source populations into account.

We have now explored several factors that could explain under-filling, and the results regarding self-powered dispersal help us to interpret the threat of further range expansion much more clearly – please see the new discussion. We felt that distance to source population might not be the most accurate way of representing the accessibility of a location. Instead we investigated both species-specific dispersal ability and the climatic continuity of the landscape through which species must navigate to reach unfilled locations.

l.117ff: I have difficulties with the textual description of regions under high threat, i.e. those

that are red/orange in Fig3. For example, I fail to see large red parts in the north-eastern US, but miss the mentioning of large parts of central and western US, and Alaska. Likewise, I do not see any red areas in Australia (maybe along the coast?) or southern South America, but no mention was made of high threats in northern Europe or Iceland.

We have now removed the climate change component of the paper

l. 154: I don't understand the argument; what has this to do with complexity of impacts? Please explain.

We have rephrased this, lines 165-166

l. 164-176: the discussion is already quite lengthy; if it needs to be shortened, I suggest to consider deleting this part of the discussions on impact magnitudes and reporting as it is not directly related to the topic of the paper

We have now completely re-written the discussion.

l. 255: add "in" after "First, "

This line has now been removed.

l. 297-301: The importance of disturbance has, to my knowledge, mainly been demonstrated in organisms that compete highly for space, i.e. sedentary species, like plants and marine benthic communities. Off the top of my head, I don't remember any study showing disturbance as being important for colonization or impact in birds or mammals. Maybe the authors can try to find some examples for the latter groups? Or discuss disturbance a bit more taxon specific?

These are good points thank you. However, re-writing the discussion has led to this section being removed.

l. 302: "invasive species are often not invasive at the first moment of introduction" is a bit contradictory. Maybe replace the first "invasive" by "alien".

This has been updated – lines 292-293.

l. 308: spell out SEICAT

The new MS does not mention SEICAT

l. 331: I am surprised that the recently published GLONAF (https://doi.org/10.1002/ecy.2542) and GAVIA (https://doi.org/10.1038/sdata.2017.41) databases were not used as I would have expected them to be the most up-to-date sources for global alien plant and bird data. I understand that data collection was carried out in 2017, but this is already 4 years ago and updated global data repositories have meanwhile been published. I think it would be very valuable to either base the paper on these data or at least to discuss how similarities of the data used here to these global databases.

We think this is an excellent point and have taken the opportunity to address it in depth. In short, we carried out a comparison of our species lists with leading datasets (GLONAF, GAVIA, DAMA) and this information is now included in the supplementary methods and Figure S13. We found the lists are at least comparable in length and spatial coverage.

Although our list of mammals and birds are shorter than the list of species known naturalised, our data would not be improved substantially by drawing from published databases on these – which only became publicly available after we had undertaken our data collection. However, our plant list is clearly much shorter than the best available dataset. Given that plants are more poorly recorded than birds or mammals, it's likely that a large proportion of the species we did not include would not have been analysable. Moreover, the within-realm geographic patterns of naturalised species in GLONAF (https://sebastian-ch.github.io/glonafAtlas/Worldview/index.html, accessed 13th April 2023) is similar to our dataset. Nonetheless, it is important to emphasize that our dataset under-represents known plant naturalisations more than bird and mammal naturalisations. We have done so in lines 269-273. These findings are part of the reason we altered the focus of the paper away from a definitive map of threat, towards assessment of why that threat has not yet been realised. So we are grateful to have the opportunity to address this

l. 364ff: I understand the reasons for the selection of species, but I think that the selection might not be representative, but biased towards more widespread species and against recent introductions. I suggest to explicitly mention and discuss the possibility of such biases and their consequences. There are also possibilities to test if the species retained in the analysis are indeed random subsets of all species introduced to an area. As a remark: just the fact that there are more alien species in an area (e.g. Europe) does not necessarily lead to a bias.

As above, we think this is a very important point, and we have explored it in depth. In summary, we investigated geographic bias by comparing species numbers in each geographic region against numbers from GLONAF (plants), GAVIA (birds), and DAMA (mammals).

We found that our dataset over-represents the relative proportions of species naturalised in the Nearctic, Palearctic, and Australasia (though not birds in Australasia, supplementary fig. S13). The discrepancy between species numbers is because we had to discard species with uncertain or very little data. It is very possible that these species have narrow native ranges and climate niches, so have small potential naturalised ranges, and ability to spread / be spread. Therefore, although we believe this issue is very important to address, we don't necessarily think our geographic analysis is as biased as the raw numbers of species might suggest. We updated our interpretation of the recorder effort results to explore this issue more extensively (lines 234-268).

l. 370: it would be helpful here to mention where (in the discussion?) and how (expert opinion or analyses) this evaluation happens in the paper

This line has been removed as the approach to tackling data bias is explained in detail elsewhere.

l.372ff: What was the reason to limit the forecasts to 3 variables? My (limited) understanding of SDMs is that the fewer variables are used the higher the chances to introduce biases by choosing variables that are not capturing enough variation in suitability. I am not a climate modeller, but are there papers on the accuracy of predictions in dependence of the number of variables in the models? Moreover, it is probably not necessary to use the same approach and variables for all taxa, as they are probably very different in their susceptibility to climate. Did you test this?

This is an important point. The general consensus is that there are no hard and fast rules for how many variables to use. Broadly speaking, using fewer variables can lead to over-generality in predictions, whereas including more variables tends to increase over-fitting, being overly-specific about the conditions species can occupy. Over-fitting particularly happens when projecting ranges between continents where seasonal combinations of climate variables differ. Over-fitting would also have been more likely for the geographic regions and large number of species with few data points. In this paper we used variables that have biologically plausible and often-observed effects on all taxa, which we feel are universally applicable. These variables have used in several other published analyses (e.g. Early and Sax 2014, Estrada, Morales-Castilla et al. 2018, Morrison, Estrada et al. 2018, Häkkinen, Hodgson et al. 2022), so our results are at least inter-comparable.

Using a single set of variables is the most common approach when constructing SDMs for large numbers of species. Using different variables for different taxa would have been problematic. First, the large number of species means it is not possible to select variables that are specifically important to each species *a priori*. Second, a model selection approach would have been strongly driven by data availability, meaning the robustness of variable selection would vary greatly between species.

In conclusion, we agree it would be preferable to construct bespoke models for each species, but this is sadly unrealistic for most analyses of large numbers of species, and doing so would have led to model-based differences between species and regions. We have therefore left the methodology as is, and hope the justification provided here and in the manuscript are sufficient.

l. 427: I think it's a very good idea to estimate recorder effort to identify uncertainties in predictions!

Thank you!

l. 449: Please define "threat from regionally naturalized species". Is this the number of alien species for which the area is suitable according to your model? Does this include only the species that are not yet naturalized?

This section has been removed from the new MS.

Reviewer #2:

The authors use a climate envelope approach to project the native climate niche of species onto their naturalised regions, suggesting that species have potential to spread much more widely than they have currently colonised. This is contrasted with national response capacity and projected to future climates.

While these results would be of wide interest, my main concern is in the validity of the global threat maps presented in Fig 1 and supporting all other figures in the main text. As detailed below, these maps likely contain major artefacts of the merging of separate assessments for each biogeographic realm, geographic bias in the selection of species and recording effort. As such I am not confident that as presented the manuscript provides a valid global estimate of regional threat from already-naturalised species.

Thank you for judging the paper to be of broad interest, and we appreciate the opportunity to address the concerns you raise by altering our approach and the MS substantially. Regarding the concern that the results do not accurately estimate threat from already-

naturalised species, we note that the major shift in emphasis now means that accurately predicting the *precise* edges of species' potential ranges is not as important as it was previously. As long as inaccuracies are minor, the key is that they do not systematically differ between taxa, regions, or explanatory variables analysed. Nonetheless, we believe the additional information and analyses we have undertaken address the methodological concerns raised.

More detailed comments are given below.

The study considers an impressive 844 species, but covers a minority of taxonomic groups (no insects or microorganisms) and only a fraction of the known number of species with naturalised populations (e.g. the Global Register of Introduced and Invasive Species lists >11000 species). In several places in the text the data are described as if they provide a more comprehensive assessment of global invasion than they likely do, which is to be avoided.

This is an important point, and part of the reason we altered the focus of the paper away from a definitive map of threat, towards assessment of why that threat has not yet been realised. We've taken the opportunity to explore our data in depth, identify any data shortfalls, and interpret any effects on our results. We carried out a comparison of our species lists with leading datasets and this information is now included in the supplementary methods and Figure S13. In short, the lists are at least comparable in length and spatial coverage.

Although our list of mammals and birds are shorter than the list of species known naturalised, our data would not be improved substantially by drawing from published databases on these – which only became publicly available after we had undertaken our data collection. However, our plant list is clearly much shorter than the best available dataset. Given that plants are more poorly recorded than birds or mammals, it's likely that a large proportion of the species we did not include would not have been analysable. Moreover, the within-realm geographic patterns of naturalised species in GLONAF (https://sebastian-ch.github.io/glonafAtlas/Worldview/index.html, accessed 13th April 2023) is similar to our dataset. Nonetheless, it is important to emphasize that our dataset under-represents known plant naturalisations more than bird and mammal naturalisations. We have done so in lines 269-273. We have added this dataset comparison to a supplementary methods section.

Unfortunately, it is currently completely impossible to look at global threat from insects, micro-organisms, and other under-recorded groups in any comprehensive way. If we attempted to do this, we would be limited to a fraction of somewhat well recorded species, which would almost certainly bias the coverage enormously towards well-studied regions of Europe and north America. We address this point in a supplementary methods section.

More importantly, the selection of species may be geographically biased towards naturalisation in particular biogeographic realms, meaning that the threat to those realms would be exaggerated in the global threat map (Fig 1). For example, it is clear from Fig S1 that the data contained very few mammal species naturalised in East Palearctic, and many in West Palearctic. As a result, Fig 1 suggests almost no threat to the East but a major threat to the West. Is this result valid, or an artefact of species selection being biased towards mammal invaders of Europe? I feel the authors need to somehow address this point by quantitatively demonstrating that their choice of species is representative of naturalisation in each realm.

Particular thanks for this comment as we agree that this is a very important issue to explore and interpret. We investigated geographic bias by comparing species numbers in each geographic region against numbers from GLONAF (plants), GAVIA (birds), and DAMA (mammals), and these results have been added to the supplementary information. Note that we had to calculate this using the geographic regions employed by each published database, so the names do not match those elsewhere in the MS, though the geographic areas are broadly the same.

As suspected, our dataset over-represents the relative proportions of species naturalised in the Nearctic, Palearctic, and Australasia (though not birds in Australasia, supplementary fig. S13). Note DAMA doesn't divide up east and west palearctic so we couldn't assess the representativeness of our mammal numbers in each region. The discrepancy between species numbers is because we had to discard species with uncertain or very little data. It is very possible that these species have narrow native ranges and climate niches, so have small potential naturalised ranges, and ability to spread / be spread. Therefore, although we believe this issue is very important to address, we don't necessarily think our geographic analysis is as biased as the raw numbers of species might suggest. We updated our interpretation of the recorder effort results to explore this issue more extensively (lines 234-268).

I strongly feel that recording effort has a major influence on the threat map in Fig. 1, including threat gradients within realms. For example, the increased apparent threat from west to east Europe for plants and mammals could easily result from massively greater recording in western Europe, as demonstrated by GBIF record density maps for Plantae and Mammalia. Indeed, the authors own investigation of this using recording effort estimates from Meyer et al (2016) demonstrate a major sensitivity to effort (Fig S2 shows very different threat maps to Fig 1). Even this attempt to accommodate effort is not ideal because it uses estimates derived at a coarser grid resolution than the present study, which could even underestimate effort biases (since species will be more likely to be recorded in a larger grid cell). I recommend the authors consider somehow using a more direct estimate of recording effort derived at the resolution of the study grid, e.g. based on the total GBIF record density for each taxonomic group.

Thank you for this comment, as we think global assessment papers too often overlook bias in recording effort. Understanding the effect of recording effort has always been central to this paper, and we believe we have taken fairly novel steps to explore these effects on our results and interpretation. The alternative threat map demonstrates the difference in invasion potential that could be caused by under-recording of naturalised species. We have edited the discussion to explore this issue as thoroughly as possible.

With respect to the approach for dealing with recorder effort, there is much evidence to suggest using the 'total GBIF record density' would be a poorer approach than the one we use. Using total GBIF records would be highly sensitive to GBIF's baked-in, systematic biases in how recorded relative abundances deviate from true natural relative abundances of species, as well as by small sample sizes in many areas (Meyer, Weigelt et al. 2016, and refs therein). For plants at least, a map produced in this way was judged less plausible than Meyer's maps, for example suggesting that some tropical regions were extremely species-poor. For further information, (Meyer, Weigelt et al. 2016) explore issues associated with recorder effort metrics extensively in SI 2.

Meyer's approach improves on simply using the 'total GBIF record density', creating an index based on the comparison between observed species richness and a relatively independent model of richness informed by species lists and environment-richness

relationships. Meyer's estimates of recorder effort are done extremely thoroughly, more so than could be done by a paper not exclusively dedicated to recorder effort. We do not believe it would be possible to come up with a more appropriate and accurate measure of recorder effort within the scope of this paper.

Finally, we agree that using a recorder effort map with a different spatial resolution to our own map is not ideal. However, it seems reasonable to assume that the recording effort of one of our 10 arc-minute grid-cell corresponds to recording effort within the Meyer's grid-cell in which it lies (for context, 10 arc-minute grid-cells are one sixth the size of Meyer's 110km cells at the equator). Issues could arise if there were geographically or taxonomically systematic differences in the correspondence, but this seems unlikely.

In my view, the climate matching approach adopted is reasonable given the large numbers of species, though some more detail is needed (e.g. was there any transformations of the climate variables such as log transform of precipitation or centring and scaling? How was the 2d kernel smoothing parameter selected?). However, as the approach is less sophisticated than other species distribution models applied to invasive species I would like to see better validation. This needs to go beyond the simple native-range validation reported to consider predictive ability in the invaded range, targeting established invaders likely to have filled most climate space. It is vital to establish that the chosen approach does not systematically overpredict potential invaded ranges.

Thank you for this assessment. We're happy to provide more detail about the methods – addition details regarding the scaling/transformation of variables have been added at 350-351. Details regarding the smoothing method have been added to L357-358 (plus a reference to the function and package used). We have tried to improve our explanation of the cross-validation we did (lines 104-111, 390-406).

We're not sure what validation against species' naturalised ranges would add? First, species are absent from most of their potential naturalised ranges so specificity will be low. However, this does not necessarily indicate that climate matching is wrong, since there are many reasons species have not filled potential naturalised ranges. Second, other work we highlight and reference in the MS (lines 262-266) demonstrates that many species undergo niche expansion in the naturalised range, further reducing specificity for some species. There are multiple reasons for niche expansion, including niche evolution, restricted native ranges, and altered biotic interactions in the naturalised realm. Without knowing which factor caused expansion into a given climate for each individual species, we cannot know the degree to which climate matching will under-predict species' ranges. Our predictions of unfilled climate space are therefore conservative. We have discussed the potential consequences of this in lines 262-266.

The major change in the MS' emphasis means that the difference between climate occupied in the native and naturalised range, i.e. the 'failure' of climate matching to fully predict current naturalised ranges, is now of central interest itself. Additionally, accurately predicting the precise edges of species' potential ranges is not as important as it was when our emphasis was the extent of the global invasion threat. Therefore, small inaccuracies in climate niche estimates are not vitally important as long as inaccuracies do not systematically differ between taxa, regions, or explanatory variables analysed.

We hope this will satisfy the reviewer that our approach is suitable for the questions we ask.

It would also be desirable to test the sensitivity of the modelling to the choice of kernel density threshold used to define suitable climates. It appears that a very generous threshold was used (1/1000 of the maximum occurrence value). This might lead to an overprediction of

the potentially invaded range by including climatic conditions very rarely occupied in the native range. Would the threat maps differ if a stricter threshold were used? For example, in niche shift studies, I believe a more conservative 70% density threshold is typically used.

This is a very good point. We have re-analysed our data using the 70% threshold suggested. The relative threat only changes in a few locations. New results are on lines 120-125, and methods are on lines 374-377.

References

Biancolini, D., V. Vascellari, B. Melone, T. M. Blackburn, P. Cassey, S. L. Scrivens and C. Rondinini (2021). "DAMA: the global Distribution of Alien Mammals database." Ecology **102**(11): e03474.

Dyer, E. E., D. W. Redding and T. M. Blackburn (2017). "The global avian invasions atlas, a database of alien bird distributions worldwide." Scientific Data **4**(1): 170041.

Early, R. and D. F. Sax (2014). "Climatic niche shifts between species' native and naturalized ranges raise concern for ecological forecasts during invasions and climate change." Global Ecology and Biogeography **23**(12): 1356–1365.

Estrada, A., I. Morales-Castilla, C. Meireles, P. Caplat and R. Early (2018). "Equipped to cope with climate change: traits associated with range filling across European taxa." Ecography **41**(5): 770-781.

Häkkinen, H., D. Hodgson and R. Early (2022). "Plant naturalizations are constrained by temperature but released by precipitation." Global Ecology and Biogeography **31**(3): 501-514.

Meyer, C., P. Weigelt and H. Kreft (2016). "Multidimensional biases, gaps and uncertainties in global plant occurrence information." Ecology Letters **19**(8): 992-1006.

Morrison, L., A. Estrada and R. Early (2018). "Species traits suggest European mammals facing the greatest climate change are also least able to colonize new locations." Diversity and Distributions **24**(9): 1321-1332.

van Kleunen, M., P. Pyšek, W. Dawson, F. Essl, H. Kreft, J. Pergl, P. Weigelt, A. Stein, S. Dullinger, C. König, B. Lenzner, N. Maurel, D. Moser, H. Seebens, J. Kartesz, M. Nishino, A. Aleksanyan, M. Ansong, L. A. Antonova, J. F. Barcelona, S. W. Breckle, G. Brundu, F. J. Cabezas, D. Cárdenas, J. Cárdenas-Toro, N. Castaño, E. Chacón, C. Chatelain, B. Conn, M. de Sá Dechoum, J.-M. Dufour-Dror, A. L. Ebel, E. Figueiredo, O. Fragman-Sapir, N. Fuentes, Q. J. Groom, L. Henderson, Inderjit, N. Jogan, P. Krestov, A. Kupriyanov, S. Masciadri, J. Meerman, O. Morozova, D. Nickrent, A. Nowak, A. Patzelt, P. B. Pelser, W.-s. Shu, J. Thomas, A. Uludag, M. Velayos, A. Verkhosina, J. L. Villaseñor, E. Weber, J. J. Wieringa, A. Yazlık, A. Zeddam, E. Zykova and M. Winter (2019). "The Global Naturalized Alien Flora (GloNAF) database." Ecology **100**(1): e02542.
