# Supplementary material for: Global terrestrial invasions: Where naturalised birds, mammals, and plants might spread next and what affects this process
Source: PLoS Biol. 2023 Nov 14;21(11):e3002361. doi: 10.1371/journal.pbio.3002361 (PMC10645288; doi:10.1371/journal.pbio.3002361)
Supplement: Attachment — Submitted filename: Response to reviewers_Jul2023_v2.docx [file pbio.3002361.s028.docx]

Response to reviewers, 3^rd^ August 2023

Dear Dr Roberts,

Thank you very much for reconsidering our manuscript, and for the positive response to our resubmission. We also greatly appreciate the attention of the academic editor and reviewer, and the very constructive suggestions we have received from all involved. We’re pleased to be able to respond to the editor and reviewer’s outstanding questions about the manuscript below.

The reviewer and editor comments are in black, and our responses in red.

Your sincerely,

Dr Henry Häkkinen, on behalf of the authors.

**FROM THE EDITOR:**

Dear Dr Early,

Thank you for your patience while we considered your revised manuscript "Global terrestrial invasion deficits and what causes them." for consideration as a Short Report at PLOS Biology. Your revised study has now been evaluated by the PLOS Biology editors, the Academic Editor and one of the original reviewers.

In light of the reviews, which you will find at the end of this email, we are pleased to offer you the opportunity to address the remaining points from the reviewers in a revision that we anticipate should not take you very long. In addition, the Academic Editor has provided some guidance which I've pasted into the foot of the email. We will then assess your revised manuscript and your response to the reviewers' comments with our Academic Editor aiming to avoid further rounds of peer-review, although might need to consult with the reviewer, depending on the nature of the revisions.

Thank you for the positive response to our revisions, we have addressed these below for your consideration.

IMPORTANT:

a) We're finding the current Title somewhat opaque, and wonder if it might be better to include more specific findings; as you say in the Abstract, "Prediction of terrestrial regions to which 833 naturalized plants, birds and mammals are most imminently likely to spread"  - could you fashion a Title more along those lines? (though I do note that you also explore the underlying drivers of this)

We have rewritten the title along the suggested lines. Our new title is “Global terrestrial invasions: where might naturalised birds, mammals, and plants spread next and what is stopping them?”. We’re always happy to take advice from editors about striking the right balance between precision and garnering broad interest with the title. So if you have any suggestions we’re happy to consider them.

b) Please address the concerns raised by reviewer #1 and the Academic Editor.

We have done so

c) Please address my Data Policy requests below; specifically, we need you to supply the numerical values underlying Figs 1ABC (note that panels are in the wrong order!), 2ABC, S1ABCDEF, S2ABC, S3ABC, S4AB, S5ABCDEF, S6ABC, S7ABC, S8ABC, S9ABCDEF, S10ABCDEF, S11ABC, S13, either as a supplementary data file or as a permanent DOI’d deposition. I note that the raw data used in your study belong to third party sources; such data are exempt from our policy as long as readers can obtain them in a similar manner; however, we will need the processed numerical values that directly underlie the Figure panels.

Apologies regarding the panel order, now corrected. We have now created a repository with all code, derived datasets and data we are permitted to share.

d) Please cite the location of the data clearly in all relevant main and supplementary Figure legends, e.g. “The data underlying this Figure can be found in S1 Data” or “The data underlying this Figure can be found in <https://doi.org/10.5281/zenodo.XXXXX>”

The links to the relevant repository are provided in the data availability statement and in the caption of each figure. The repository can be found at https://doi.org/10.5281/zenodo.8205905 and there is a full description of how and where to access the data underlying each figure in the ReadMe

Thank you again for your submission to our journal. We hope that our editorial process has been constructive thus far, and we welcome your feedback at any time. Please don't hesitate to contact us if you have any questions or comments.

Sincerely,

Roli Roberts

Roland Roberts, PhD
Senior Editor
PLOS Biology
[rroberts@plos.org](mailto:rroberts@plos.org)

----------------------------------------------------------------

REVIEWERS' COMMENTS:


Reviewer #1:

PBIOL-D-23-01216.R1

The revision has improved the paper a lot and I also like the new focus. The authors appropriately justify their methods (but see below). My comments on the revision are generally minor, if the statistical approach chosen by the authors holds up to scrutiny and results do not change if a different approach would be chosen.

Thank you very much for this feedback.

I'd like the authors to consider three general issues in their revision:

First, the description of which parameters have been tested in each taxon (plants, birds, mammals) was sometimes unclear and should be carefully revised. I noticed some obvious errors below in the detailed comments, but the entire section would profit from a careful revision.

Thank you for your feedback on this, we have revised several sections as the reviewer suggests, please see our responses below. In particular we have now added a supplementary table which details all parameters trialled in the Bayesian hierarchical model.

Second, the model selection strategy (step-wise procedures; reliance on one "best" model) chosen by the authors has been criticised a lot in the statistical literature. Although in my experience results from such analyses are often very similar to those from recommended "superior" analyses (e.g. comparison of all plausible models; inference considering multiple models), I think the paper would be strengthened if the authors would justify their choice of statistical procedure. Otherwise, a switch to less contested methods might be more appropriate, but it is difficult to estimate how much this would change the outcomes.

On re-reading this section, I agree we do not justify our approach very well. We have re-written this section (Lines 553-576) to make our approach clearer. In short, we could not build a model with all variables at once as it would cause huge convergence issues. Instead, we created univariate models to “scan” for variables of interest and then built a multivariate model with those variables. If convergence issues continued with that full set of variables, we removed the least important variable until the multivariate model converged. We have now listed all multivariate models trialled in the supplementary material (table s10-12). We selected the best model(s) for the results (as described in the methods), which conveniently for birds and mammals was a single clear best model. For plants, there were two nearly equally performing models, and we have now included the results from these models in the supplementary materials. Our results or selected models have not changed from the previous version of the manuscript.

Finally, I find it unfortunate that the authors do not make the data available by claiming not to be the property holders of the data. Thus, it will be close to impossible to re-run the analyses to confirm the integrity of the results.

I agree this is unfortunate and we have tried to make as much available as possible. We have now created a static repository of the code (https://doi.org/10.5281/zenodo.8205905), which we now reference in the manuscript. In this repository we have also provided our derived datasets where possible, and dummy datasets where not so readers can trial the methodology without needing to download data. We have included the readme file for this repository in the resubmission.

Going through the analysis step-by-step:

1) The PCA used to model species potential naturalised ranges. We provide the code to do this. This uses GBIF occurrence data which we cannot share directly with third parties as many of the data sources have usage restrictions (<https://www.gbif.org/terms/data-user>). Instead, we provide a link in the manuscript to the derived dataset we created and used in this analysis which can be downloaded directly from the GBIF website. This provides a full list of sources and details of occurrence data used. In addition, in our online repository we have provided some dummy data for plant species so that readers can run the PCA code we provide and inspect the integrity of the results.

2) The output from the PCA includes shapefiles of each species estimated native and naturalised ranges, rasters of niche unfilling and expansion, diagnostic plots, and climate-matching validation statistics. We are happy to share these, but the data are around 100GB and not easily interpretable. We have chosen not to upload these but they are available on request and we have made this clear in the manuscript (lines 601).

3) The above outputs are condensed into taxon-specific rasters of invasion deficit. We have provided these in the repository.

4) Validation and sensitivity analysis of climate matching. We provide the code files to do this.

5) Sensitivity to recorder effort. We provide the code files to do this.

6) We also provide all code to make GIS maps and figures.

7) The Bayesian hierarchical analysis of factors affecting range filling. This is based on key outputs from the PCA (which are provided) and a number of species traits. We calculated habitat fragmentation ourselves, and provide this in the repository. However other trait data were derived directly from external data sources which cannot be shared with 3^rd^ parties. These sources are all provided in the manuscript and additional details are provided in the repository. In the meantime, we have provided dummy sets of variables to allow users to run and explore the code effectively.

We hope that by providing the code, links to the GBIF dataset, and dummy data we can at least illustrate our methods and make analysis using the same methods more replicable.

Detailed comments:

Table 1: please harmonise whether WAIC (legend) or DIC (table) was used. We have done this

l. 198: maybe give references for the effect of these traits in affecting invasion (e.g. some of these traits have been shown to affect establishment success in both birds and mammals) Have now added reference to support this point (see line 202).

l. 204: interesting hypothesis; is this independently supported in the literature? To our knowledge this has not been investigated extensively, but Rolland and Salamin (2016) did find that on average, bird thermal niches were broader than other taxonomic groups. We have added a note (line 208) and appropriate reference.

l. 224ff: This argument would also apply to other taxa, but here you seem to have found evidence mainly for birds. Could you discuss this? This is a good point. We had highlighted birds in Florida as the most extreme example of unexplained range-unfilling. However, these arguments could certainly apply to other groups in other contexts. We have clarified this point (lines 234-236)

l. 359: add reference Apologies, we have now added this (line 377).

l. 368: delete "they" done (line 386)

l. 433: ALA = Atlas of Living Australia? Yes, we have added full name for clarity (line 451)

l. 452-8: It is unclear to me if this applies to all taxa (plants, birds, mammals) or only to some. Please specify. E.g. the reference for brain size only applies to mammals, but brain size information is also available for birds (reference 55 in the paper). We have clarified the point. We did not use brain size for birds due to lack of data for the species we studied (line 476-478).

l. 469: for mammals? Yes, we have now made this clear in MS (line 489)

l. 482: are dominant Corrected (line 503)

l. 487: "their overall abundance"? please clarify to what this refers We have added additional detail (line 508).

l. 521ff: The model selection process follows a step-wise strategy, which I guess is at least partly due to the computational complexity of conducting a comparison of models with all possible combinations of factors, or a comparison of all "plausible" models (i.e. those that could be defended without knowing the results). Although in my experience step-wise model selection and comparison of all possible models often yields very similar results, the step-wise selection process has been widely criticised in the literature (see references below) and I think that some justification for this approach would convince readers of the robustness of the findings and leave authors less exposed to criticism. Similarly, relying on one "best-fitting" model is usually considered as inferior to drawing inference over all credible models (e.g. by model averaging); also this choice would profit from more justification.

We think we may have poorly explained our process, it was meant more that if in doubt we chose a parsimonious model and our use of the term “step-wise selection” was wrong in this case. What we meant was we made a single ‘fullest’ model containing all parameters except those that co-varied strongly or prevented model convergence. We then made and tested several other more parsimonious models to see if we could improve model performance. We have re-written this section extensively (see lines 553-576), please see our response to your earlier comment regarding this for more details.

l. 529: delete (pD)? pD was correct in the original context, but following re-writes this has now been removed.

Figure 1: please arrange letters a,b,c from top to bottom Apologies for this, we have now corrected.

Figure 1: there is a strong divide in the number of species that could expand their alien ranges across the Ural Mountains in all 3 taxa and I wonder if this might be an artefact e.g. due to the choice of separating east and west Palaearctic or recording bias (although the authors seem to rule out the latter). I didn't see in the map any other transition of similar strength, thus I'd like the authors to discuss this and particularly why this is the same in all taxa.
This is a good observation, and it is due to our division of the palearctic into east and west. We have added text in the discussion to discuss this artefact (see lines 273-280), and recommend that the invasion deficit just east of the Urals may be higher than depicted.

Anderson, D. R., & Burnham, K. P. (2002). Avoiding pitfalls when using information-theoretic methods. The Journal of wildlife management, 66, 912-918.

Burnham, K. P., Anderson, D. R., & Huyvaert, K. P. (2011). AIC model selection and multimodel inference in behavioral ecology: some background, observations, and comparisons. Behavioral ecology and Sociobiology, 65, 23-35.

Whittingham, M. J., Stephens, P. A., Bradbury, R. B., & Freckleton, R. P. (2006). Why do we still use stepwise modelling in ecology and behaviour?. Journal of animal ecology, 75(5), 1182-1189.

COMMENTS FROM THE ACADEMIC EDITOR [lightly edited]:

The revision done in this version has been exhaustive and the authors did a good job. The three issues pointed out by reviewer #1 require further revision, however.

A small table in Suppl Mat describing in detail the parameters used in each group could be very useful and clarify very much the overall approach.

Thank you for this suggestion, it is now included (see Table S8)

The authors need to carefully consider the model selection approach. Stepwise procedures have been critiqued recently and most recent approaches to model selection are done with modern model-combination techniques (e.g., multi-model averaging, etc.). Please see, e.g.: Whittingham et al. 2006 J Animal Ecol. [https://doi.org/10.1111/j.1365-2656.2006.01141.x](https://protect-eu.mimecast.com/s/FCg6CQ754i67JrghGjUDW?domain=doi.org)

In brief, stepwise selection procedures have three main weaknesses: bias in parameter estimation, inconsistencies among model selection algorithms, and an inappropriate focus or reliance on a single "best model". I'd recommend the authors to carefully consider an improved approach to their model selection strategy.

Thank you for bringing this up, I think our original manuscript was in error as although we did tend towards a more parsimonious model when all else was equal, we did not follow a true stepwise procedure. We have rewritten this section extensively to clarify our process, and have also included results from alternative models (two of which were equally viable for plants) in the supplementary material. Please see our notes in response to reviewer 1 for additional details, and lines 553-576 in the MS for an improved explanation of the process.

I also agree with the issue about data availability. This is a must nowadays, especially for this type of compilation paper. PLOS Biology has detailed instructions and requirements about data availability.
We apologise for this, but we are not permitted to reshare the occurrence and trait data used throughout this study. However, the data can be downloaded from the original online repositories. We have created a repository with all the relevant code, data we are allowed to share, and links to download data we cannot share directly. We have linked to this in the data accessibility section. Where we cannot include original data, we have created a dummy dataset to allow researchers to run and explore our methods. We believe we have provided all data and code necessary for our results to be reproduced if the reader downloads occurrence and trait data from the original repositories. We hope this is sufficient to meet your policy, which we have tried to meet to the best of our ability as data accessibility and reproducibility is very important to us.
